# Strategies for enhancing the representation of women in clinical trials: an evidence map

**DOI:** 10.1186/s13643-023-02408-w

**Published:** 2024-01-02

**Authors:** Karen M. Goldstein, Lindsay Chi Yan Kung, Susan Alton Dailey, Aimee Kroll-Desrosiers, Colleen Burke, Megan Shepherd-Banigan, Rebecca Lumsden, Catherine Sims, Julie Schexnayder, Dhara Patel, Sarah Cantrell, Kate L. Sheahan, Jennifer M. Gierisch

**Affiliations:** 1 Center of Innovation to Accelerate Discovery and Practice Transformation, Durham Veterans Affairs Health Care System, 508 Fulton Street, Durham, NC 27705 USA; 2 Division of General Internal Medicine, Duke University School of Medicine, 40 Duke Medicine Circle, Durham, NC 27710 USA; 3 Health Management & Policy, Graduate School of Public Health, San Diego State University, 5500 Campanile Drive, San Diego, CA 92182-4162 USA; 4 VA Central Western Massachusetts Healthcare System, 421 North Main Street, Leeds, MA 01053 USA; 5 UMass Chan Medical School, 55 Lake Ave. N, Worcester, MA 01655 USA; 6 Department of Population Health Sciences, Duke University School of Medicine, 215 Morris Street, Durham, NC 27701 USA; 7 Duke-Margolis Center for Health Policy, 100 Fuqua Drive, Box 90120, Durham, NC 27708 USA; 8 Duke Rheumatology Division, 40 Duke Medicine Circle Clinic 1j, Durham, NC 27710 USA; 9 University of Alabama at Birmingham School of Nursing, NB545 1720 2nd, Ave S, Birmingham, AL 35294 USA; 10 Duke University Medical Center Library & Archives, Duke University School of Medicine 10 Searle Drive, Durham, NC 27710 USA; 11 JSI, 2733 Crystal Drive, Arlington, VA 22202 USA

**Keywords:** Women, Clinical trials, Representation, Participation

## Abstract

**Background:**

Equitable sex- and gender-based representation in clinical trials is an essential step to ensuring evidence-based care for women. While multi-institutional actions have led to significant improvements in the inclusion of women in trials, inequity persists in areas like sex-neutral cancers and cardiovascular disease. We sought to identify strategies described or evaluated to boost the inclusion of women in clinical trials.

**Methods:**

We used evidence mapping methodology to examine the breadth of relevant literature. We developed an *a priori* protocol and followed reporting guidance from the Preferred Reporting Items for Systematic Reviews and Meta-Analysis where applicable. We searched MEDLINE^®^ (via PubMed) and EMBASE (via Elsevier) databases from inception through April 4, 2023, and used standardized procedures incorporating duplication and data verification. We included articles that described strategies to improve the recruitment and retention of women in clinical trials.

**Results:**

We identified 122 articles describing recruitment and retention strategies for 136 trials (377,595 women). Only one article distinguished between the sex and gender identity of participants, and none defined their use of the terms such as “women” or “female”. The majority of articles (95%) described recruitment for only women, and 64% were conducted in the USA. Ninety-two articles (75%) described strategies in the context of sex-specific conditions (e.g., gynecologic diagnosis). The majority of included articles evaluated a behavioral intervention (52%), with 23% evaluating pharmacologic interventions and 4% invasive interventions. The most common trial phase for reported strategies was during outreach to potential participants (116 articles), followed by intervention delivery (76), enrollment (40), outcomes assessment (21), analysis and interpretation (3), and dissemination (4). We describe specific types of strategies within each of these phases.

**Conclusions:**

Most of the existing literature describing strategies to improve the inclusion of women draws from trials for sex-specific conditions and is largely related to outreach to potential participants. There is little information about how and if studies have attempted to proportionally increase the inclusion of women in trials with both men and women or those focused on invasive and pharmacologic interventions. Future work in this area should focus on how to increase the participation of women in mixed-sex studies and on those areas with remaining inequities in trial participation.

**Supplementary Information:**

The online version contains supplementary material available at 10.1186/s13643-023-02408-w.

## Introduction

Equitable representation by sex and gender in clinical trials is an essential step to ensuring true patient-centered, evidence-based care. An individual’s sex, a determination made at birth based on an individual’s biology, and gender, a construct based on an individual’s lived social and cultural experiences, each has the potential to influence the effect of an intervention or natural history of an illness. In the current era of precision medicine and personalized medical care, failure to incorporate an evidence-based understanding of the influence of sex and gender on an individual’s health represents a missed opportunity to optimize outcomes and risks an adverse health event. For example, women experience much higher rates of adverse medication side effects related to sex-based differences in pharmacokinetics, yet for most commonly used medications, the original clinical trials supporting their approval did not report sex-stratified outcomes [1]. In order to be able to generate sex and gender-specific science, we need sufficient numbers of women in trials to support the statistically sound exploration of differential treatment effects.

Recent decades have seen the implementation of multi-national legislation to overcome the historical discriminatory exclusion of women from trials [2]. In the 30 years, since the NIH established the Office of Research on Women’s Health (ORWH), multiple programs have spurred new investigations into women’s health, funding to support the career development of women investigators, and the development of a rich array of tools to support the inclusion of sex and gender in clinical research [3]. In 1998, the FDA established a mandate to include both men and women as well as sex-based analyses for trials supporting the approval of drugs intended for both sexes [4]. While actions like these have led to improvements and near parity in sex-based participation in many drug trials [5], inequity persists in important areas. In particular, women continue to be underrepresented in trials related to cancer [6], chronic kidney disease, vascular disease [7], and certain cardiovascular conditions [8–12].

To increase the proportion of women participating in clinical research, evidence-based strategies are needed to enhance the inclusion of women in trials. Prior work has explored how certain study design features (e.g., blinded intervention assignment) influence trial participation rates generally [13], but we know less about what other trial design features (e.g., population engagement in trial design) and study conduct approaches (e.g., gender concordant study staffing) have been deployed to increase participation by women specifically. In particular, understanding how study teams have strategically approached trial activities with the purpose of promoting adequate representation of women could inform future trials as they attempt to address participation disparities. Specifically, we sought to develop a broad understanding of where there is evidence about approaches deployed to include women across the lifespan of a trial. Thus, we conducted an evidence map to answer the following question: *What strategies have been described or evaluated to boost the enrollment or retention of women in clinical trials?*

## Methods

We selected evidence mapping methodology as it is appropriate for reviews that seek to describe the breadth of a body of literature and identify areas for future research rather than focus on the specific effects of a narrow, defined intervention [14, 15]. We were unable to find a prior review assessing approaches to include women in trials, thus starting with developing a broad understanding was appropriate. We developed an *a priori* protocol that was posted online: (https://osf.io/cbhxt?view_only=cd368b067f2644869b504a83d156fae6) and followed reporting guidance from the Preferred Reporting Items for Systematic Reviews and Meta-Analysis (PRISMA) guidelines where applicable (e.g., we did not evaluate risk of bias or estimate overall effect).

To support clarity, we established definitions for key terms (see Additional file 1). Of note, while we planned to report both sex and gender as presented within included studies, we found that the current literature did not report these constructs separately nor did they routinely define their use of terms such as “women”, “female”, “sex”, or “gender”. We acknowledge that this is conceptually problematic as it conflates the distinct dimensions of sex and gender. Due to this limitation of the existing primary literature, we use the term “women” to reflect any individuals reported by an article to be women or female from this point on. We note that this challenge has implications for research on both sex-based and gender-based differences [16].

### Search strategy

We searched MEDLINE^®^ (via PubMed) and EMBASE (via Elsevier) databases from inception to 4/4/2023. An experienced medical librarian (SC) devised and conducted the searches, with input on keywords from the other authors. We used a combination of database-specific subject headings and keywords related to women, recruitment, retention, and clinical trials. Editorials, letters, case reports, and comments were excluded. To increase specificity, pediatric-only literature was also excluded. The searches were independently peer-reviewed by an additional librarian using a modified PRESS Checklist [17]. The full, reproducible search strategies for all included databases are located in Additional file 2. In addition, we reviewed the references of previous systematic reviews conducted on related topics for potentially relevant references [18–21].

### Study selection

We used prespecified eligibility criteria for both quantitative or descriptive (Additional file 3) and qualitative (Additional file 4) articles. Articles describing recruitment or retention strategies employed during the conduct of a clinical trial with the intent to increase the inclusion of women and which targeted participants, study staff, or investigators were eligible. We excluded articles describing trials conducted outside of countries recognized by the Organization for Economic Co-operation and Development (OECD) [22] and those not available in English. Article titles and abstracts were reviewed by all co-authors (except SC) for potential relevance to the research question with one vote leading to inclusion for full-text review and two for exclusion. At the full-text screening, pairs of investigators agreed on the final article disposition status. Disagreements were resolved by consensus or by obtaining a third reviewer’s opinion (KMG). Covidence (Veritas Health Innovation, Melbourne, Australia), a web-based software that streamlines literature and systematic reviews, was used for screening and tracking screened and included articles [23].

Given the small number of relevant articles describing recruitment and retention outcomes, we also included those with narrative descriptions of strategies in a trial that did not include specific outcomes of interest or used a study design not appropriate to evaluate strategies (i.e., non-comparative designs). Given the large number of articles identified describing trials, we prioritized these over self-identified pilot studies or feasibility trials. We considered the unit of analysis to be the article rather than the trial as some articles described strategies used across more than one trial. Additional file 5 lists articles excluded at the full-text review stage and the reasons for exclusion.

### Data abstraction

Data abstraction was conducted by two sub-teams: one focused on study characteristics and the second on described strategies impacting recruitment and/or retention. The first sub-team (SAD, JS, CB, RL, DP, CS) abstracted high-level study characteristics using a form developed in Covidence for data abstraction. Study characteristic abstraction was piloted with each reviewer abstracting data from 6 articles in round 1 of piloting and 10 articles in round 2. Abstracted characteristics included information to provide context for study strategies such as patient descriptors (e.g., age, sex/gender, race/ethnicity), intervention characteristics being studied in the trial (e.g., disease condition, invasiveness of intervention), trial design (e.g.*,* comparator, study setting), stated recruitment goals, and final recruitment numbers.

The second sub-team (AKD, LCK, KMG, KS, MSB, DP, CS) abstracted study-specific recruitment and retention strategies into a REDCap form [24]. To pilot this form, each reviewer was assigned the same 4 articles to pilot for the first round and 20 articles for the second round. After each round of piloting with both groups, results were compared and discussed, and the abstraction forms were modified. Subsequently, data from each included article was abstracted by at least one reviewer. Twenty percent of each reviewer’s abstractions were over-read to verify accuracy. For those reviewers whose abstraction quality was considered insufficient based on the frequency of errors of either commission or omission, the entirety of that reviewer’s data abstractions were over-read and corrected as needed. We approached strategy abstraction by trial phase: trial development (e.g., patient and community partner engagement, trial staff training), participant outreach (e.g., location, modality, and partners for potential participant outreach), enrollment (e.g., flexible modality and location for consent), intervention delivery (e.g., flexible timing for intervention delivery) and outcomes assessment (e.g., remote data collection), analysis (e.g., recruitment/retention by gender/sex), and dissemination (e.g., plans to share trial results with participants or community).

### Assessment of methodological quality of individual articles

As this is an evidence mapping review, we did not assess the methodological quality of individual articles or conduct certainty of evidence ratings.

### Data synthesis

We narratively summarized the study characteristics of the identified literature using relevant data abstracted from the eligible articles. We organized trial strategies by trial phase as described above and then by level of targeted action (e.g., study participant, source community, study team). We then looked for patterns across articles related to types of recruitment/retention strategies employed in relationship to characteristics such as type of intervention (e.g., pharmacologic vs. behavioral), whether the condition studied was sex-specific or not (e.g., pregnancy vs. cardiovascular disease), and population studied. In particular, we considered reporting patterns of specific strategies by articles focused on the recruitment of women from historically marginalized racial/ethnic populations. Descriptive statistics about the included studies were calculated in Microsoft Excel [25].

## Results

**Fig. 1:**
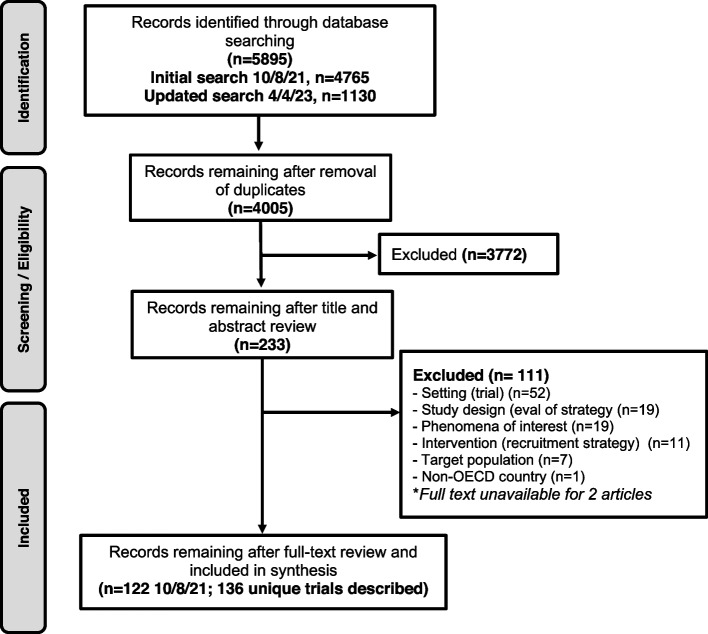
Literature flow

We identified 122 eligible articles (see Fig. 1) reporting on recruitment and retention strategies for 136 trials (total *n* = 377,595 women; median 285 per trial). Of the 122 articles, 95% recruited only women. Seven articles reported on trials that recruited participants other than women: 1 recruited couples, 3 recruited men and women (not couples), and 3 recruited women and children. The majority of articles reflected trials conducted in the USA (64%). Fifty-eight articles provided narrative descriptions of strategies employed, 72 provided some degree of comparison between strategies used either within a study or between studies, and 10 articles reported qualitative data collection from either trial participants or study staff about trial recruitment experiences (see Additional file 6 for a detailed description of included articles). Ninety-two (75%) of all articles addressed sex-specific conditions (e.g., peripartum conditions, gynecologic conditions) vs. 30 (25%) that addressed sex-neutral conditions (e.g., infectious diseases, cardiovascular disease). Few articles were relevant to conditions recognized to have an evidence base that underrepresents women; for example, we found only 6 articles describing strategies in cardiovascular trials and none relevant to sex-neutral cancers or chronic kidney disease. Behavioral interventions were most common (52%), followed by pharmacologic (23%), and only 4% were invasive in nature. The majority of the pharmacologic and invasive interventions were for sex-specific conditions (20 of 28 and 4 of 5, respectively). Forty-one articles described strategies for purposefully recruiting women from a specific racial or ethnic group with Black or African-American being the most common (*n* = 19) and 14 articles reported on strategies to include multiple racial/ethnic minoritized populations (Table 1).

**Table 1:** Summary of included articles describing recruitment strategies for women

**Article characteristics**	***n* = number of articles unless otherwise indicated**
Total number articles included (#trials described)	122 (136)^a^
Total number of women recruited (total *N*; median; range per study)	377,595^b^; 285; (1–202,638)^c^
Total number of articles reporting an *a priori* recruitment target	52
Population recruited	
Women only	116
Couples only	1
Women and children	2
Men or women (individually)	3
Site of recruitment efforts by country	
United States	78
United Kingdom	13
Australia	11
Canada	6
>1 Country	7
Other	7
Methodologic approach^d^	
Descriptive	58
Compared strategies	72
Primary qualitative	10
Target condition for trial (women-specific)	
Peripartum	37
Cancer	27
Menopause	10
Gynecologic conditions	9
General women’s health	3
Urogynecologic	3
Cancer prevention	1
Contraception	1
Infectious diseases	1
Target condition for trial (not women-specific)	
Metabolic health	7
Infectious disease	6
Bone health	4
Cardiovascular disease	4
Mental health	3
Nutrition	2
Cancer survivorship	1
Interpersonal violence	1
Not specified	1
Partner health	1
Intervention type^d^	
Behavioral	63
Invasive	5
Pharmacologic	28
Multiple	13
Other	13
Virtual intervention component	28
Recruitment/retention strategies described by phase^e^	
Trial development	56
Potential participant outreach	116
Enrollment/consent process	40
Intervention delivery	76
Outcomes assessment	21
Analysis and interpretation	3
Dissemination	4
Race or ethnic group of interest	
Asian	2
Black or African American only	19
Latina only	5
Native Hawaiian or other Pacific Islander only	1
Multiple racial/ethnic minoritized populations	14
No focus on recruiting a specific racial or ethnic group	81
Rural/urban	
Urban populations	15
Rural populations	9
Age groups^f^	
Studies that include participants <18 years	7
Total age range (years)	12–70
Studies that include participants >50 years	38*

^a^One hundred seventeen articles, 131 trials described, 4 articles did not specify the number of trials, and 2 articles described the same trial

^b^Of the 6 studies that did not exclusively recruit women, 1 article did not report the number of women recruited separately from the total

^c^Eight studies did not report a number of women recruited

^d^Categories are not mutually exclusive

^e^Number of studies reporting at least one strategy

^f^Studies that include participants >50 include 17 studies that also include participants younger than 50 (e.g., 18–65)

**Fig. 2:**
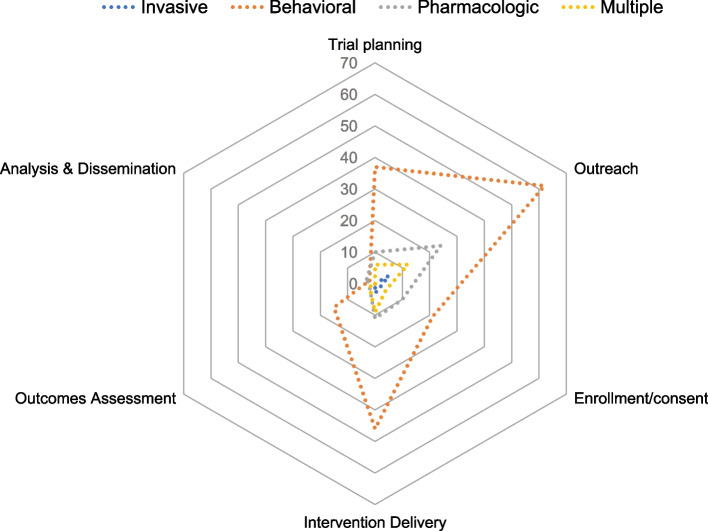
Studies with at least one strategy in a study phase, by intervention type

**Fig. 3:**
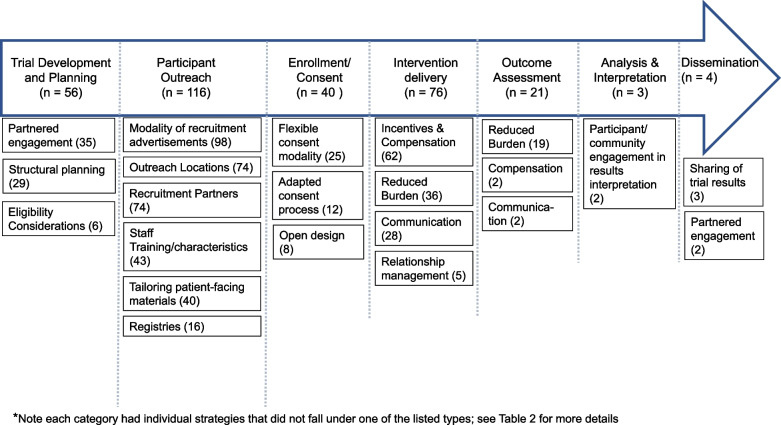
Strategy types across trial phases (*n* = number of articles)*

Described strategies were most commonly reported during the stage of a trial in which the study team conducts outreach to potential participants (95%; Tables 2, 3, 4, 5, 6, 7 and 8). Outreach was the most predominant stage across all intervention types (Fig. 2). The most common strategy for this trial stage was the modality of recruitment advertisements which was reported in 98 articles (e.g., mass media, print letters, word of mouth), identification of community partners in the recruitment process in 74 articles (e.g., clinicians, laypersons, or peers), followed by the location for outreach efforts reported in 74 articles (e.g., clinics, community-based locations, churches), and tailoring of patient-facing recruitment materials by 40 articles (e.g., tailoring the language or images, or developing bilingual materials) (Fig. 3).

**Table 2:** Detailed strategies by trial stage: Trial Planning (56 articles)

Strategies reported during trial planning	# Articles
**Partnered engagement during trial design (35 articles**^a^**)**	
Community/patient engagement	18
Clinician engagement	12
Intentional community relationship building	6
Budget allocation for partner engagement	76
CBPR	17
**Structural planning (29 articles)**	
Plan to monitor retention	15
Continuous recruitment method refinement	3
Site selection consideration	12
Theory-based recruitment planning	11
**Eligibility considerations (6 articles)**	
Intentional inclusion of reproductive age women	3
Pragmatic eligibility criteria	3
**Other**^b^** (7 articles)**	
Co-design of recruitment plan with recruitment staff, cross-site recruitment planning, piloting of recruitment approach, budgeting for trial material translation, and ensuring all participants receive intervention	

^a^Each article may have reported more than one strategy within each subcategory

^b^Each strategy listed under “other” was mentioned by one study

**Table 3:** Detailed strategies by trial stage: Participant outreach (116 articles)

Strategies reported during potential participant outreach	# Articles
**Staff training/characteristics (43 articles)**	
Race/ethnicity concordant staff	20
Sex/gender concordant staff	12
Bilingual staffing	9
Communication training	6
Race/ethnicity (awareness?) training	4
Sex/gender awareness training	1
Trauma-informed care training	1
Other unspecified staff training	20
**Location for outreach efforts (74 articles)**	
Clinic (e.g., women’s health clinic, primary care, inpatient service)	51
Community locations (e.g., housing projects, senior centers, schools, women-only fitness centers, hair salon, library, breastfeeding support group)	51
Churches	28
Health fairs	20
**Partners for the recruitment process (74 articles)**	
Clinicians	61
Peer/laypersons	30
Support from a community leader/organization	3
Public relations agency consultation	3
Community partner referrals	2
Payment to community partners for referrals	2
**Modality of recruitment advertisements (98 articles)**	
Public advertisement (posters, flyers, bus ads)	59
Direct contact (emails, letters, texting)	57
Mass Media (newspaper, magazines, radio, TV, newsletters, PSA)	60
Online/social media/mobile app	35
Word of mouth	24
Group information sessions	26
Direct outreach to clinicians	27
Toll-free hotline	3
**Registries (16 articles)**	
Use of disease-specific registry	12
Public registries	4
**Tailoring of patient-facing recruitment materials (40 articles)**	
Message tailoring	35
Image choice	14
Participant testimonial including	5
Bilingual materials	8
**Other**^**a**^**(8 articles)**	
Automatic medical record algorithm, communication training for referring clinicians, access to an interpreter, study logo on swag, creation of outreach toolkit, existing list-serves, dedicated study phone line, campus electronic sign, hand-writing letters, reallocation of funding for advertisement	

^a^Each strategy listed under “other” was mentioned by one study

**Table 4:** Detailed strategies by trial stage: Enrollment (40 articles)

Strategies reported during enrollment	# Articles
Flexible consent modality (Virtual consent only, home, asynchronous, choice of location, online screening, flexible timing)	25
Adapted consent forms or process	12
Open/Zelen design	8
**Other**^a^ **(7 articles)**	
private space for consenting, choice of treatment arm, extra time to complete processes, consent waiver, screening at community clinic, real-time scheduling, convenient site access	

^a^Each strategy listed under “other” was mentioned by one study

**Table 5:** Detailed strategies by trial stage: Intervention delivery (76 articles)

Strategies reported during intervention delivery	# Articles
**Incentives & compensation (62 articles)**	
Financial or material incentives (include food)	56
Childcare availability/reimbursement	8
Transportation reimbursement (parking validation,)	14
Educational credit	1
Connect to other health resources/services	1
Entertainment	1
**Reduced intervention burden (36 articles)**	
Flexibility (timing, location)	28
Remote modality of intervention delivery	13
Limited intervention complexity to reduce burden	4
**Communication (28 articles)**	
Reminders to engage	23
Ongoing Study communications (e.g., Birthday cards, newsletter, postcards, thank you notes Other	7
Sharing of interim results	3
**Relationship management (5 articles)**	
Intentional relationship building	2
Complaint follow up	1
Frequent contact	1
Study staff continuity	1

**Table 6:** Detailed strategies by trial stage: Outcomes assessment (21 articles)

Strategies reported during outcomes assessment	# Articles
**Compensation (2 articles)**	
Childcare availability/reimbursement	2
Transportation reimbursement	1
**Reduced assessment burden (19 articles)**	
Flexible outcomes assessment (modality, timing	12
Limited burden (incl passive, remote, time, location)	10
**Communication (2 articles)**	
Reminder	2

**Table 7:** Detailed strategies by trial stage: Analysis/interpretation (3 articles)

Strategies reported during analysis/interpretation	# Articles
Participant/community engagement in results interpretation	2

**Table 8:** Detailed strategies by trial stage: Dissemination results (4 articles)

Strategies reported during dissemination results	# Articles
Partners assisted with dissemination	2
Trial results provided to participants	3

The next most common trial phase was intervention delivery (62% of all included articles); specific strategies in this category included incentives and compensation (62 of 76 articles mentioning intervention delivery strategies), reduced intervention burden (36 articles), communication (28 articles), and study staff-participant relationship management (5 articles). Strategies relevant to trial planning were reported by 56 articles. Common groups of strategies were relevant to partnered engagement in trial design, structural planning (e.g., site selection considerations), and eligibility considerations. Forty articles reported strategies related to the enrollment process including flexibility around the consent process (e.g., modality, timing), adapted consent process, and open design. Strategies relevant to outcomes assessment were reported by 21 articles and were primarily related to reducing the assessment burden. Two articles mentioned the involvement of participant and community member engagement in results interpretation, 3 articles provided final trial results to participants, and 2 engaged with partners around dissemination.

When considering articles focused on intersecting identities, we found that articles about recruiting women from marginalized racial/ethnic populations more often reported strategies during the planning phase of trials (78%) and intervention delivery (75%) compared to articles without a specific population subfocus (30% and 55%, respectively). Finally, we considered strategies across the trial phase across both sex-specific and non-sex-specific conditions (Additional file 7). We found across all conditions that the majority of strategies fell in the first 3 trial phases (i.e., trial development, outreach, enrollment).

## Discussion

The peer-reviewed literature evaluating and/or describing strategies to boost the inclusion of women in clinical trials is drawn primarily from trials of sex-specific conditions and most commonly described strategies pertaining to the process of outreach to potential participants. A smaller proportion of included articles discussed strategies used for recruiting women in trials evaluating invasive interventions or medications and almost exclusively focused on sex-specific conditions. There were notably fewer strategies described during study conceptualization and design, analysis and interpretation, or dissemination. Additionally, there was almost no literature describing approaches to increase the participation of women within trials that included both men and women, and very little in trials targeting conditions known to persistently underrepresent women (e.g., cardiovascular disease, sex-neutral cancers). Finally, only one article made the explicit distinction between sex and gender among participants identified as women.

Prior work on trial recruitment provides context for our findings albeit through a sex and gender-neutral lens. In an umbrella review by Rodriguez-Torrez et al. [26], barriers and facilitators to trial participation were described across 20 distinct themes. Most relevant included concerns about intervention characteristics (e.g., risk of side effects or time burden), personal obstacles to participation (e.g., transportation, childcare, work schedules), need for information about the trial (e.g., clarity and messaging about trial participation requirements), and the influence of others on decisions to participate (e.g., friends, family, institutions). While their analysis was not stratified by sex or gender, the barriers noted by Rodriguez-Torrez and colleagues are typically more common among women due to the typical gendered distribution of social responsibilities related to childcare and reliance on social support for decision making. Many of the strategies that we identified were directly relevant to these established barriers (e.g., compensation for childcare, relationship management during trial interactions).

A second prior review by Treweek et al. [13] reported on 68 eligible trials exploring different design features and their impacts on trial recruitment, though they also did not include a sex-specific analysis. They noted 3 design features whose impact was found to have a high certainty of evidence, including open vs. blinded/placebo trials, telephone reminders to those who do not respond to postal invitations, and using a bespoke approach to developing participant information materials drawing on population-specific input. While telephone reminders are a popular recruitment approach for targeting underrepresented populations, as illustrated by its appearance in 38 of our articles reviewed, this approach was found to be ineffective by Treweek et al. It is possible that population-specific tailoring of study materials is more effective among certain groups. For example, women Veterans were more likely to participate in a survey study when approached using “enhanced recruitment materials” designed to build trust through pictures of the study team and detailed information about their skills and experience [27].

There is also overlap with our findings among study teams aiming to enhance the participation of other specific patient populations. Bonevski et al. [28] conducted a review of recruitment strategies targeting socially disadvantaged populations. They describe many similar strategies to those we identified including population-tailoring of study materials, use of alternative sampling strategies (e.g., snowball, targeted, oversampling); community-engaged approaches such as community outreach, patient collaborators input on intervention design research and recruitment, incentives; flexibility in data collection (e.g., timing and/or modality); and use of bilingual materials. Obtaining the endorsement of community leaders and engagement of community members to provide culturally congruent expertise has also been employed to boost population-specific representation [29].

One challenge to enhancing the inclusion of women in trials is that women as a population are not a homogenous community. An individual who identifies as a woman may or may not have been assigned ‘female’ at birth (i.e., Cis-gender) and will bring her own intersectional collection of identities to the research setting. We found no studies that identified participants by both sex and gender nor any that specified recruitment goals across these constructs. Collecting and reporting sex and gender identity is critical to support future work to understanding how recruitment approaches might be tailored to ensure sex and gender parity in trial participation. Fortunately, many of the identified strategies that promote flexibility and ease of participation will likely benefit all potential participants and could be applied as universal design principles. For example, while potentially more common among women, barriers such as caregiving responsibilities [30], unemployment, and transportation problems [31], could be addressed by identified strategies such as flexible intervention delivery modality and minimal data collection burden.

Women are as likely, or more likely, to participate in research as men when given the opportunity [32–34] which may explain why the majority of strategies identified in this review were around potential participant outreach. However, study retention also warrants attention. Prior work on retention among women in research has identified ongoing remote contact as a helpful strategy [35], along with the importance of interactions with study staff [28, 31]. Purposeful trust-building and establishing a positive-caring rapport within participant-study staff relationships is an important strategy for other historically marginalized populations within research and minoritized populations are often willing to participate despite underlying distrust [36, 37]. Interestingly, we found relatively little around the specific training of study staff or the establishment and promotion of the trial staff-participant relationship. Only one study described sex and/or gender awareness training, four reported race/ethnicity-focused training, and only 2 noted intentional relationship building with participants. Other approaches to staff training could include engendering a trust-worthy study environment through the incorporation of trauma-informed care principles (TIC). TIC was first developed within the context of mental health treatment as an approach to normalize the individual reaction to a traumatic experience and focus on a strengths-based approach to recovery [38]. TIC is now being incorporated into multiple clinical settings including virtual primary care [39, 40].

In addition to being the first review to focus solely on strategies employed to boost the representation of women, we also considered strategies across the entire lifespan of a clinical trial. Our approach adds to that of Bonevski et al. who expanded their perspective on trial phases beyond outreach and trial design but did not consider analysis or dissemination. Accordingly, the next steps in the inclusion of women in clinical trials will need to involve a comprehensive and *a priori*-defined approach to the deployment of strategies across the lifespan of the trial. Work from the Collaborative Institutional Training Program (CITI) has emphasized the importance of “upstream” recruitment planning and incorporated this approach into their recruitment framework [41]. In fact, among the most effective efforts to boost inclusion in the National Lung Screening Trial were setting an a priori recruitment goal for individuals from minoritized populations and planning trial recruitment efforts in advance [42]. An example of a population-specific tool for recruitment and retention can be found in the “5Ts” framework for the inclusion of older adults [43] which outlines key steps to ensuring that clinical studies are accommodating to the needs of older adults (e.g., allowing more time and tips to accommodate).

Strengths of this work include using a standardized, rigorous, and *a priori-*defined protocol; however, our findings should be considered within the context of its limitations. First, we approached this review as an evidence map to describe the breadth of literature related to the topic of enhancing the representation of women in trials; therefore, in keeping with this review approach, we did not conduct a quality assessment of the identified literature or propose specific conclusions about the most impactful strategies for the inclusion of women. However, we have included the author’s reported conclusions in an additional file as a reference for future analysis (see Additional file 9). We note that similar to work with other targeted populations [28, 44], there were few trials directly comparing strategies which will limit the drawing of inferences about strategy effectiveness. This reality reflects the dearth of efficacious evidence-driven approaches to boost gender diversity in recruitment, further signaling a need for higher-level exploration of the differences between genders and sexes [42]. A second limitation was the variability in the definition of “women” among authors, which often conflated “gender” and “sex,” making it difficult to accurately identify approaches boosting gender diversity among recruitment. In addition, we did not evaluate if the strategies described would be applicable to gender-diverse persons as well as who may be susceptible to sex-specific conditions (e.g., ovarian cancer). This blurs the important distinction between sex and gender which is an important area for future work. When possible, we identified when a single study was described across multiple articles; however, it is possible that we missed some such studies due to a lack of reported detail in the included articles. Finally, we describe strategies as written by the investigators from individual trials. We suspect that there were likely actions taken to enhance the representation of women but which were not documented. In particular, this is possibly related to analysis and dissemination that may have been mentioned in main results articles from relevant trials but not included in manuscripts related to recruitment and retention if not conceptualized as relevant.

## Conclusion

The research community has called for equitable representation of women in clinical trials to ensure the generalizability of scientific evidence and to inform sex-specific, evidence-based care. Strategies to enhance the representation of women in clinical research need to be considered across the lifespan of a trial to promote long-term participant engagement stemming from investment and trust-building beyond the initial signing of a consent form. While many such strategies have been used to date, very few have been described in the context of non-sex-specific research (e.g., cardiovascular disease, cancer). The persistent representation gap in these critical areas of clinical research limits their generalizability and stands in the way of patient-centered, evidence-based care for women. Future work should explore which strategies are most effective to ensure the appropriate participation of women in clinical research on conditions relevant to both men and women.

### Supplementary Information

**Additional file 1: Appendix.** Definitions.

**Additional file 2: Appendix 2.** Search Strategies.

**Additional file 3: Appendix 3.** Eligibility Criteria for Quantitative Studies.

**Additional file 4: Appendix 4.** Eligibility Criteria for Qualitative Studies.

**Additional file 5: Appendix 5.** Articles Excluded at Full-Text Review Stage, with Reasons.

**Additional file 6: Appendix 6.** Characteristics of Included Articles.

**Additional file 7: Appendix 7.** Articles with a focus on participants from racial or ethnic minoritized populations.

**Additional file 8: Appendix 8.** Strategies reported by trial phase across women-specific and non-specific conditions.

**Additional file 9: Appendix 9. **Author-Reported Conclusions from Included Articles.

## Data Availability

The datasets used and/or analyzed during the current study are available from the corresponding author on reasonable request.
