# Supplementary material for: Strategies for enhancing the representation of women in clinical trials: an evidence map
Source: Syst Rev. 2024 Jan 2;13:2. doi: 10.1186/s13643-023-02408-w (PMC10759390; doi:10.1186/s13643-023-02408-w)
Supplement: Supplementary file 1 — Additional file 1: Appendix. Definitions. [file 13643_2023_2408_MOESM1_ESM.docx]

**Appendix 1. Definitions**

To guide this review, we adopted the following definitions:

**Trial**: “A research study in which one or more human subjects are prospectively assigned to one or more interventions (which may include placebo or other control) to evaluate the effects of those interventions on health-related biomedical or behavioral outcomes.” ^1^

**Recruitment strategy**: “A recruitment intervention was defined as any method implemented to improve the number of participants recruited to a randomized controlled trial, whether this was directed at potential participants, at those responsible for recruiting participants or at trial design or co-ordination.” ^2^ We extended this definition of recruitment strategies to apply to retention strategies as well given our interest in overall representation. We conceptualized retention strategies as those approaches described to initially support participation by women through intervention delivery and primary outcome collection.

**Sex: “**A biological variable defined by characteristics encoded in DNA, such as reproductive organs and other physiological and functional characteristics”^3^

**Gender**: “Refers to social, cultural, and psychological traits linked to human males and females through social context.”^3^

**References**

1. NIH's Definition of a Clinical Trial. 2017. (Accessed March 22, 2023, at <https://grants.nih.gov/policy/clinical-trials/definition.htm>.)

2. Treweek S, Pitkethly M, Cook J, et al. Strategies to improve recruitment to randomised trials. The Cochrane database of systematic reviews 2018;2:Mr000013. doi: 10.1002/14651858.MR000013.pub6.

3. National Academies of Sciences, Engineering, and Medicine. (2022). Measuring sex, gender identity, and sexual orientation. [White paper]. The National Academies Press; <https://doi.org/10.17226/26424>
