# Supplementary material for: Strategies for enhancing the representation of women in clinical trials: an evidence map
Source: Syst Rev. 2024 Jan 2;13:2. doi: 10.1186/s13643-023-02408-w (PMC10759390; doi:10.1186/s13643-023-02408-w)
Supplement: Supplementary file 2 — Additional file 2: Appendix 2. Search Strategies. [file 13643_2023_2408_MOESM2_ESM.docx]

**Appendix 2. Search Strategies**

Librarian searcher: Sarah Cantrell, MLIS; Duke University Medical Center Library & Archives; Duke University School of Medicine

Peer review of search conducted by: Brandi Tuttle, MLIS; Duke University Medical Center Library & Archives; Duke University School of Medicine

**Database: MEDLINE (via PubMed)**

Original search date: 6/30/2021
Search update: 4/4/2023

| **Search Set** | **Search Strategy** | **Results 6/30/21** | **Results 4/4/23** |
| --- | --- | --- | --- |
| #1  *women* | "Women"[Mesh] OR women*[tiab] OR woman*[tiab] OR female*[tiab] OR gender-specific[tiab] OR gender-tailored[tiab] | 2,155,740 | 2,412,078 |
| #2  *recruitment* | "Patient Selection"[Mesh] OR "patient recruitment"[tiab] OR "recruiting patients"[tiab] OR "patient enrollment"[tiab] OR "enrolling patients"[tiab] OR "patient accruement"[tiab] OR "accruing patients"[tiab] OR "patient selection"[tiab] OR "selecting patients"[tiab] OR "patient volunteer"[tiab] OR "patient volunteers"[tiab] OR "subject recruitment"[tiab] OR "recruiting subjects"[tiab] OR "subject enrollment"[tiab] OR "enrolling subjects"[tiab] OR "subject accruement"[tiab] OR "accruing subjects"[tiab] OR "subject selection"[tiab] OR "selecting subjects"[tiab] OR "subject volunteer"[tiab] OR "subject volunteers"[tiab] OR "patient retainment"[tiab] OR "retaining patients"[tiab] OR "subject retainment"[tiab] OR "retaining subjects"[tiab] OR underrepresent*[tiab] | 109,933 | 120,275 |
| #3  *Clinical Trials* | "Clinical Trials as Topic"[Mesh] OR "Therapeutic Human Experimentation"[Mesh] OR trial[tiab] OR trials[tiab] OR "human experiment"[tiab] OR "human experiments"[tiab] OR "human experimentation"[tiab] OR "intervention study"[tiab] OR "intervention studies"[tiab] | 1,308,023 | 1,478,091 |
| #4  *combining* | #1 AND #2 AND #3 | 2,867 | 3,266 |
| #5  *Study design exclusions* | #4 NOT (Editorial[pt] OR Letter[pt] OR Case Reports[pt] OR Comment[pt]) | 2,783 | 3,172 |
| #6  *Pediatric literature exclusion* | #5 NOT (("Adolescent"[Mesh] OR "Child"[Mesh] OR "Infant"[Mesh]) NOT "Adult"[Mesh]) | 2,712 | 3,083 |
| Search Update | #6 AND ("2021/01/01"[Date - MeSH] : "3000"[Date - MeSH]) | n/a | 577 |

**Database: EMBASE**Original search date: 6/30/2021
Search update: 4/4/2023
*Note: search from the Results page*

| **Search Set** | **Search Strategy** | **Results 6/30/21** | **Results 4/4/23** |
| --- | --- | --- | --- |
| #1 *women* | women*:ti,ab OR woman*:ti,ab OR female*:ti,ab OR 'gender specific':ti,ab OR 'gender tailored':ti,ab | 3,104,117 | 3,605,741 |
| #2  *recruitment* | 'Patient Selection'/exp OR 'patient recruitment':ti,ab OR 'recruiting patients':ti,ab OR 'patient enrollment':ti,ab OR 'enrolling patients':ti,ab OR 'patient accruement':ti,ab OR 'accruing patients':ti,ab OR 'patient selection':ti,ab OR 'selecting patients':ti,ab OR 'patient volunteer':ti,ab OR 'patient volunteers':ti,ab OR 'subject recruitment':ti,ab OR 'recruiting subjects':ti,ab OR 'subject enrollment':ti,ab OR 'enrolling subjects':ti,ab OR 'subject accruement':ti,ab OR 'accruing subjects':ti,ab OR 'subject selection':ti,ab OR 'selecting subjects':ti,ab OR 'subject volunteer':ti,ab OR 'subject volunteers':ti,ab OR 'patient retainment':ti,ab OR 'retaining patients':ti,ab OR 'subject retainment':ti,ab OR 'retaining subjects':ti,ab OR underrepresent*:ti,ab | 140,808 | 166,637 |
| #3  *Clinical Trials* | 'clinical trial (topic)'/exp OR 'therapeutic research'/exp OR trial:ti,ab OR trials:ti,ab OR 'human experiment':ti,ab OR 'human experiments':ti,ab OR 'human experimentation':ti,ab OR 'intervention study':ti,ab OR 'intervention studies':ti,ab | 1,754,519 | 2,032,669 |
| #4  *combining* | #1 AND #2 AND #3 | 3,227 | 4,449 |
| #5  *Study design exclusions* | #4 NOT ('case report'/exp OR 'case study'/exp OR 'editorial'/exp OR [editorial]/lim OR 'letter'/exp OR [letter]/lim OR 'note'/exp OR [note]/lim OR [conference abstract]/lim OR 'conference abstract'/exp OR 'conference abstract'/it) | 2,064 | 2,664 |
| #6  *Pediatric literature exclusion* | #5 NOT (([child]/lim OR [infant]/lim OR [newborn]/lim OR [preschool]/lim OR [school]/lim) NOT ([adult]/lim OR [middle aged]/lim OR [young adult]/lim)) | 2,035 | 2,628 |
| Search update | #6 AND [01-01-2021]/sd | n/a | 550 |
