# Supplementary material for: Strategies for enhancing the representation of women in clinical trials: an evidence map
Source: Syst Rev. 2024 Jan 2;13:2. doi: 10.1186/s13643-023-02408-w (PMC10759390; doi:10.1186/s13643-023-02408-w)
Supplement: Supplementary file 3 — Additional file 3: Appendix 3. Eligibility Criteria for Quantitative Studies. [file 13643_2023_2408_MOESM3_ESM.docx]

# Appendix 3. Eligibility Criteria for Quantitative Studies

| **Study Characteristic** | **Inclusion Criteria** | **Exclusion Criteria** |
| --- | --- | --- |
| Population | - Trial participants - Adults (18 years +) identified (by self or as per electronic medical record) as women; - Trial staff – individuals responsible for recruitment into a study - Trial investigators – individuals responsible for studies which aim to enhance participation of women   Studies that focus on subpopulations of women (e.g., minoritized women) are eligible | - Trial participants who are children or populations less than 75% adults identified as women |
| Intervention | Strategies employed during the conduct of a trial for the express purpose of recruiting, enrolling, and/or retaining women participants   - Strategies can be delivered at the patient, study team, or system level - Strategies can target individual potential trial participants, study team members or the system in which research takes place | Recruitment or retention strategies used by studies that may have included women but did not explicitly seek to enhance the inclusion of women  Studies which examine the impact of changes to eligibility criteria only or adjustments to sampling frame |
| Comparator | Any or none | NA |
| Outcome | - Number and/or proportion of women consented, enrolled, drop-out or retention rates (e.g., number of women completing final outcome assessment) - Recruiting to *a priori* identified recruitment target for women - Measures of subjective experiences with strategies as completed by potential participants trial staff or trial investigators | Number or proportion consented, enrolled, drop-out or retention rates *not* reported by gender  Measures of subjective experience with recruitment or retention activities if not specific to strategies intended to enhanced inclusion of women |
| Timing | No limit | NA |
| Setting | (Note that this refers to the trial in which a recruitment enhancement strategy is used)   - Any trial type (randomized, feasibility) which is employing defined strategies intended to enhance the recruitment of women - Trials that include women only and which employ strategies explicitly intended for enhancement recruitment based on participant identity as women (e.g. tailoring recruitment activities to gathering spaces for women’s groups) - Trials that include any gender, but which employ strategies explicitly to enhance the recruitment of women   Trials can be evaluating interventions for any condition | Studies not meeting the below definition of trial  Studies without prospective recruitment, observational, or other prospective study designs that do not involve participant assignment to an intervention  Trials that do not clearly describe strategies to promote enhancement of recruitment and retention of women  Hypothetical trials |
| Study design | (Note this refers to the study evaluating or describing the use of the recruitment enhancement strategy in a trial)  EPOC   - Randomized trials - Nonrandomized trials - Controlled before-after studies - Interrupted time series   Observational   - Cohort   Relevant systematic reviews  Descriptive papers that detail strategies used to intentionally enhance inclusion of women in a specific trial but which do not include evaluation (eg, protocol papers)  Pilot/feasibility studies which include a comparison of at least 2 different recruiting strategies or describe a novel recruitment strategy designed to enhance the inclusion of women (Note comparison could be historical, sequential, or parallel). | - Editorial, nonsystematic review, letter to the editor, conference abstract) - Clinical guidelines - Individual patient case study   Studies that examine associations between individual-level participants characteristics and enrollment status (comparing those who enroll vs. those who do not).  Protocol papers which may describe recruitment strategies as part of an overall protocol description but which do not specifically describe approaches which were designed to enhance the inclusion of women. |
| Language | Any | Any |
| Years | Any | NA |
| Countries | OECD^a^ | Non-OECD |
| Publication types | Full publication in a peer-reviewed journal | Letters, editorials, reviews, dissertations, meeting abstracts |

^a^ OECD = Organization for Economic Co-operation and Development includes Australia, Austria, Belgium, Canada, Chile, Czech Republic, Denmark, Estonia, Finland, France, Germany, Greece, Hungary, Iceland, Ireland, Israel, Italy, Japan, Korea, Latvia, Luxembourg, Mexico, Netherlands, New Zealand, Norway, Poland, Portugal, Slovak Republic, Slovenia, Spain, Sweden, Switzerland, Turkey, United Kingdom, United States
