# Supplementary material for: Strategies for enhancing the representation of women in clinical trials: an evidence map
Source: Syst Rev. 2024 Jan 2;13:2. doi: 10.1186/s13643-023-02408-w (PMC10759390; doi:10.1186/s13643-023-02408-w)
Supplement: Supplementary file 4 — Additional file 4: Appendix 4. Eligibility Criteria for Qualitative Studies. [file 13643_2023_2408_MOESM4_ESM.docx]

# Appendix 4. Eligibility Criteria for Qualitative Studies

| **Study Characteristic** | **Inclusion Criteria** | **Exclusion Criteria** |
| --- | --- | --- |
| Setting | (Note that this refers to the trial in which a recruitment enhancement strategy is used)   - Any trial type (randomized, feasibility) which is employing defined strategies to enhance the recruitment of women - Trials that include women only and which employ strategies explicitly intended for enhancement recruitment based on participant identity as women (e.g. tailoring recruitment activities to gathering spaces for women’s groups) - Trials that include any gender, but which employ strategies explicitly to enhance the recruitment of women - Trials can be evaluating interventions for any condition | Studies not meeting the below definition of trial  Studies without prospective recruitment, observational, or other prospective study designs that do not involve participant assignment to an intervention  Trials that do not clearly describe strategies to promote enhancement of recruitment and retention of women  Hypothetical trials. |
| Perspective | Individuals responsible for recruiting women into trials, OR,  Women participants recruited into trials | NA |
| Intervention/  Phenomena of Interest | Strategies employed to enhance recruitment and retention of women into trials with a focus on the study processes | Strategies intended to enhance recruitment not specific to women  Personal reasons why eligible participants do or do not participate in a specific clinical trial  Barriers and facilitators to trial participation generally without a specific focus on recruitment and/or retention |
| Comparison | None | NA |
| Evaluation | Experiences with strategies aimed to enhance recruitment or retention of women participants in trials | NA |
| Language | Any | Any |
| Years | Any | NA |
| Countries | OECD^a^ | Non-OECD |
| Publication types | Full publication in a peer-reviewed journal | Letters, editorials, reviews, dissertations, meeting abstracts, protocols without reporting outcomes of interest |
