# Supplementary material for: Strategies for enhancing the representation of women in clinical trials: an evidence map
Source: Syst Rev. 2024 Jan 2;13:2. doi: 10.1186/s13643-023-02408-w (PMC10759390; doi:10.1186/s13643-023-02408-w)
Supplement: Supplementary file 5 — Additional file 5: Appendix 5. Articles Excluded at Full-Text Review Stage, with Reasons. [file 13643_2023_2408_MOESM5_ESM.docx]

Appendix 5. Articles Excluded at Full-Text Review Stage, with Reasons

| **Author, year** | **Title** | **Exclusion Reason** |
| --- | --- | --- |
| Abhyankar 2016 | Identifying components in consent information needed to support informed decision making about trial participation: An interview study with women managing cancer | Setting |
| Andersen 2005 | Using a population-based cancer registry for recruitment of newly diagnosed patients with ovarian cancer | Setting |
| Andrighetti 2017 | Women's experiences of participating in a prospective, longitudinal postpartum depression study: insights for perinatal mental health researchers | Setting |
| Atiomo 2009 | Local recruitment experience in a study comparing the effectiveness of a low glycaemic index diet with a low calorie healthy eating approach at achieving weight loss and reducing the risk of endometrial cancer in women with polycystic ovary syndrome (PCOS) | Setting |
| Avis 2006 | Factors associated with participation in breast cancer treatment clinical trials | Intervention |
| Baker 2005 | Factors that influence women's decisions about whether to participate in research: an exploratory study | phenomena of interest |
| Battistella 2010 | Evaluation of methods and costs associated with recruiting healthy women volunteers to a study of ovulation | Setting |
| Bento 2008 | Process for obtaining informed consent: women's opinions | Non-OECD country |
| Bevel 2018 | Sistas Inspiring Sistas Through Activity and Support (SISTAS): Study Design and Demographics of Participants | Phenomena of interest |
| Beydoun 2006 | Combining conditional and unconditional recruitment incentives could facilitate telephone tracing in surveys of postpartum women | Setting |
| Bieniasz 2003 | Women's feedback on a chemopreventive trial for cervical dysplasia | Setting |
| Boonzaier 2010 | The practical challenges of recruitment and retention when providing psychotherapy to advanced breast cancer patients | Study design |
| Braun 2015 | Research on U.S. Military Women: Recruitment and Retention Challenges and Strategies | Study design |
| Brennan 2020 | A qualitative study of the factors influencing recruitment to a pilot trial on the prevention of striae gravidarum | Study design |
| Bringuier 2022 | Enrollment of older metastatic breast cancer patients in first-line clinical trials: 9-year experience of the large-scale real-life multicenter French ESME cohort | Phenomena of interest |
| Brown 2000 | Challenges of recruitment: focus groups with research study recruiters | Setting |
| Brown-Peterside 2001 | Retaining hard-to-reach women in HIV prevention and vaccine trials: Project ACHIEVE | Setting |
| Cadmus Bertram 2011 | Feasibility of institutional registry-based recruitment for enrolling newly diagnosed breast cancer patients in an exercise trial | Setting |
| Cambron 2004 | Recruitment and accrual of women in a placebo-controlled clinical pilot study on manual therapy | Study design |
| Chang 2009 | Participant recruitment and retention in a pilot program to prevent weight gain in low-income overweight and obese mothers | Study design |
| Close 2016 | Factors Affecting Recruitment and Attrition in Randomised Controlled Trials of Complementary and Alternative Medicine for Pregnancy-Related Issues | Intervention |
| Cooley 2003 | Challenges of recruitment and retention in multisite clinical research | Setting |
| Courvoisier 2022 | Facilitators and barriers of women's participation in HIV clinical research in Switzerland: A qualitative study | Setting |
| Coward 2002 | Partial randomization design in a support group intervention study | Study design |
| Daley 2008 | Feasibility of an exercise intervention for women with postnatal depression: a pilot randomised controlled trial | Study design |
| DeShields 2020 | Building Partnerships and Stakeholder Relationships for HIV Prevention: Longitudinal Cohort Study Focuses on Community Engagement | Setting |
| Drenkard 2020 | Overcoming barriers to recruitment and retention of African-American women with SLE in behavioural interventions: lessons learnt from the WELL study | Setting |
| Du 2009 | An educational video to increase clinical trials enrollment among breast cancer patients | Setting |
| Dubé 2020 | Participant Perspectives in an HIV Cure-Related Trial Conducted Exclusively in Women in the United States: Results from AIDS Clinical Trials Group 5366 | Study design |
| Dubé 2021 | Considerations for Increasing Racial, Ethnic, Gender, and Sexual Diversity in HIV Cure-Related Research with Analytical Treatment Interruptions: A Qualitative Inquiry | Phenomena of interest |
| Ellis 2002 | Informing breast cancer patients about clinical trials: a randomized clinical trial of an educational booklet | Setting |
| Foldi 2022 | Clinical Outcomes and Immune Markers by Race in a Phase I/II Clinical Trial of Durvalumab Concomitant with Neoadjuvant Chemotherapy in Early-Stage TNBC | Intervention |
| Fredman 2009 | Quantifying the recruitment challenges with couple-based interventions for cancer: applications to early-stage breast cancer | Intervention |
| Goldkind 2010 | Enrolling pregnant women in research--lessons from the H1N1 influenza pandemic | Setting |
| Greenfield 2014 | Implementing substance abuse group therapy clinical trials in real-world settings: challenges and strategies for participant recruitment and therapist training in the Women's Recovery Group Study | Target population |
| Grewe 2016 | Women in HIV cure research: multilevel interventions to improve sex equity in recruitment | Setting |
| Guthrie 2019 | Facebook advertising for recruitment of midlife women with bothersome vaginal symptoms: A pilot study. | Study design |
| Haley 2014 | Venue-based recruitment of women at elevated risk for HIV: an HIV Prevention Trials Network study | Setting |
| Haste 2018 | Problems recruiting and retaining postnatal women to a pilot randomised controlled trial of a web-delivered weight loss intervention | Study design |
| Herbell 2019 | Using Facebook To Recruit Pregnant Women for Research | Setting |
| Howard 2009 | Methodological challenges in evaluating the effectiveness of women's crisis houses compared with psychiatric wards: findings from a pilot patient preference RCT | Study design |
| Huang 2013 | ResearchTracking: Monitoring gender and ethnic minority recruitment and retention in cancer symptom studies | Intervention |
| Jackson 2003 | Research staff turnover and participant adherence in the Women's Health Initiative | Intervention |
| Johnson 2003 | Retention of under-served women in clinical trials: a focus group study | Intervention |
| Joseph 2007 | Recruiting low-income healthy women to research: an exploratory study | Setting |
| Juraskova 2008 | Improving informed consent: pilot of a decision aid for women invited to participate in a breast cancer prevention trial (IBIS-II DCIS) | Setting |
| Kelly 1996 | Recruitment of women into research studies: a nursing perspective | Study design |
| Kinney 1998 | The effect of physician recommendation on enrollment in the Breast Cancer Chemoprevention Trial | Setting |
| Kira 2016 | Recruiting Pregnant Indigenous Women Who Smoke into a High Contact Incentivized Cessation Trial: A Feasibility Study | Setting |
| Knight 2010 | Barriers encountered when recruiting obese pregnant women to a dietary intervention | Setting |
| Korde 2009 | Recruitment to a physical activity intervention study in women at increased risk of breast cancer | Setting |
| Kuroki 2010 | Addressing clinical trials: can the multidisciplinary Tumor Board improve participation? A study from an academic women's cancer program | Setting |
| Lawton 2017 | Written versus verbal consent: a qualitative study of stakeholder views of consent procedures used at the time of recruitment into a peripartum trial conducted in an emergency setting | Study design |
| Lawton 2016 | Recruiting and consenting into a peripartum trial in an emergency setting: a qualitative study of the experiences and views of women and healthcare professionals | Phenomena of interest |
| Le 2022 | Improving African American women's engagement in clinical research: A systematic review of barriers to participation in clinical trials | Study design |
| Lewis 1998 | Recruitment strategies in the women's health trial: feasibility study in minority populations. WHT:FSMP Investigators Group. Women's Health Trial:Feasibility Study in Minority Populations | Setting |
| Lie 2019 | Should I stay or should I go? A qualitative study exploring participation in a urology clinical trial | Phenomena of interest |
| Macleod 2013 | Recruiting and retaining postpartum women from areas of social disadvantage in a weight-loss trial--an assessment of strategies employed in the WeighWell feasibility study | Setting |
| Maghera 2014 | You are how you recruit: a cohort and randomized controlled trial of recruitment strategies | Setting |
| Manders 2014 | Factors associated with clinical trial screening failures in gynecologic oncology | Intervention |
| Maslin-Prothero 2006 | The role of the multidisciplinary team in recruiting to cancer clinical trials | Setting |
| McCarthy-Keith 2010 | Recruitment and retention of women for clinical leiomyoma trials | Setting |
| McFarlane 2007 | Strategies for successful recruitment and retention of abused women for longitudinal studies | Setting |
| Merz 2022 | Clinical trials-related knowledge, attitudes, and behaviors among Black and Latina women: A randomized controlled trial of the Women United: Clinical Trials and the Fight Against Breast Cancer Program | Setting |
| Middleton 2017 | Preventing recurrence of endometriosis by means of long-acting progestogen therapy (PRE-EMPT): report of an internal pilot, multi-arm, randomised controlled trial incorporating flexible entry design and adaption of design based on feasibility of recruitment | Study design |
| Miller 2023 | Recruitment of diverse community health center patients in a pragmatic weight gain prevention trial | Target population |
| Mohanna 1999 | Withholding consent to participate in clinical trials: decisions of pregnant women | Phenomena of interest |
| Mouton 1997 | Barriers to black women's participation in cancer clinical trials | Phenomena of interest |
| Nápoles 2014 | Randomized controlled trial of Nuevo Amanecer: a peer-delivered stress management intervention for Spanish-speaking Latinas with breast cancer | Study design |
| Neill 1998 | Recruitment and retention of women in nontherapeutic clinical trials | Phenomena of interest |
| Oberoi 2022 | Documenting patients' and providers' preferences when proposing a randomized controlled trial: a qualitative exploration | Setting |
| Osann 2011 | Recruitment and retention results for a population-based cervical cancer biobehavioral clinical trial | Phenomena of interest; |
| Penckofer 2011 | Improving subject recruitment, retention, and participation in research through Peplau's theory of interpersonal relations | Study design |
| Phillips 2011 | Recruiting Latina families in a study of infant iron deficiency: a description of barriers, study adjustments and review of the literature | Intervention |
| Resnick 2003 | Recruitment of older women: lessons learned from the Baltimore Hip Studies | Phenomena of interest |
| Ridley-Merriweather 2022 | A novel qualitative approach for identifying effective communication for recruitment of minority women to a breast cancer prevention study | Setting |
| Riggan 2023 | "There's not enough studies": Views of black breast and ovarian cancer patients on research participation | Setting |
| Rimel 2015 | A novel clinical trial recruitment strategy for women's cancer | Setting |
| Robinson 2001 | The use of e-mail in the identification of bulimia nervosa and its treatment | Setting |
| Rogers 2002 | Effectiveness of media strategies to increase enrollment and diversity in the Women's Health Registry | Setting |
| Rogers 2007 | Recruitment of women research participants: the Women's Health Registry at the University of Michigan | Setting |
| Sadler 2006 | Health parties for African American study recruitment | Setting |
| Samayoa 2022 | Cortisol levels in rural Latina breast cancer survivors participating in a peer-delivered cognitive-behavioral stress management intervention: The Nuevo Amanecer-II RCT | Intervention |
| Schonfeld 2009 | Women and contraception in research: a pilot study | Setting |
| Sedhai 2022 | Heart failure clinical trial enrollment at a rural satellite hospital | Target population |
| Sisk 2008 | The success of recruiting minorities, women, and elderly into a randomized controlled effectiveness trial | Target population |
| Smith 2018 | Sistas Taking a Stand for Breast Cancer Research (STAR) Study: A Community-Based Participatory Genetic Research Study to Enhance Participation and Breast Cancer Equity among African American Women in Memphis, TN | Setting |
| Smith 2007 | Perceptions of clinical research participation among African American women | Setting |
| Smyth 2012 | Deciding to join a perinatal randomised controlled trial: experiences and views of pregnant women enroled in the Magpie Trial | Phenomena of interest |
| Spada 2021 | Recruitment of adults with moderate eczema for a randomised trial: Comparison of traditional versus modern methods | Target population |
| Stanton 2013 | Responding to a significant recruitment challenge within three nationwide psychoeducational trials for cancer patients | Target population |
| Strömmer | Improving recruitment to clinical trials during pregnancy: A mixed methods investigation | Phenomena of interest |
| Sugarman 1999 | Ethical ramifications of alternative means of recruiting research participants from cancer registries | Setting |
| Sutton 2017 | Strategies for Successful Recruitment of Pregnant Patients into Clinical Trials | Setting |
| Timmermans 2009 | Should endometrial polyps be removed in patients with postmenopausal bleeding? -an assessment of study designs and report of a failed randomised controlled trial (ISRCTN73825127) | Phenomena of interest |
| Tincello 2009 | Colposuspension or TVT with anterior repair for urinary incontinence and prolapse: results of and lessons from a pilot randomised patient-preference study (CARPET 1) | Study design |
| Tooher 2008 | A thematic analysis of factors influencing recruitment to maternal and perinatal trials | Setting |
| Trivedi 2022 | Lessons from the Failure to Complete a Trial of Denosumab in Women With a Pathogenic BRCA1/2 Variant Scheduling Risk-Reducing Salpingo-Oophorectomy | Setting |
| Unson 2001 | The effects of knowledge, attitudes, and significant others on decisions to enroll in a clinical trial on osteoporosis: implications for recruitment of older African-American women | Phenomena of interest |
| vandenBrink 2020 | Factors affecting patient recruitment to trials: qualitative research in general practice | Phenomena of interest |
| Verghese 2021 | Willingness of postmenopausal women to participate in a study involving local vaginal oestrogen treatment as an adjunct to pelvic organ prolapse surgery: a qualitative study | Phenomena of interest |
| Welton 1999 | Is recruitment more difficult with a placebo arm in randomised controlled trials? A quasirandomised, interview based study | Setting |
| Winhusen 2012 | The potential impact of recruitment method on sample characteristics and treatment outcomes in a psychosocial trial for women with co-occurring substance use disorder and PTSD | Intervention |
| Wood 2016 | Recruitment and Participation of Older Lesbian and Bisexual Women in Intervention Research | Setting |
| Wragg 2000 | Information presentation and decisions to enter clinical trials: A hypothetical trial of hormone replacement therapy | Setting |
| Zhao 2018 | Factors influencing the recruitment of lactating women in a clinical trial involving direct oral anticoagulants: a qualitative study | Phenomena of interest |
