# Supplementary material for: Strategies for enhancing the representation of women in clinical trials: an evidence map
Source: Syst Rev. 2024 Jan 2;13:2. doi: 10.1186/s13643-023-02408-w (PMC10759390; doi:10.1186/s13643-023-02408-w)
Supplement: Supplementary file 6 — Additional file 6: Appendix 6. Characteristics of Included Articles. [file 13643_2023_2408_MOESM6_ESM.docx]

**Appendix 6. Characteristics of Included Articles**

| **Author, Year, Country, DOI** | **Recruitment strategy evaluation methods** | **Trial Condition** | **Trial Target population*** | **Trial Intervention** | **Trial phases with described recruitment strategy** |
| --- | --- | --- | --- | --- | --- |
| Agnew 2013, UK  DOI: 10.1177/1740774512460144 | Comparative | Urinary Incontinence [urogynecology] | Women 60 years of age or older, community dwelling; no population focus | 60-minute interactive workshop on continence self-management delivered in group format to 8-16 participants by trained continence nurse compared to group lecture on health issues for older women (Behavioral) | Trial Development; Outreach; Enrollment/consent |
| Akers 2018, US; Canada  DOI: 10.2196/jmir.9372 | Descriptive | Smokeless tobacco use [partner health] | Female partners of male smokeless tobacco users; no population focus | Female partners of male smokeless tobacco users were taught support skills via website and printed guidebook. This group was compared to a delayed treatment control group. (Behavioral) | Outreach; Enrollment/consent; Trial Engagement; Outcomes Assessment |
| Albrecht 2013, US  DOI: 10.1016/j.apnr.2013.05.003 | Descriptive | Ovarian cancer [sex-specific cancer] | Women between 18 and 75 years with stage III or IV disease; no population focus | 3 months of complementary and alternative medicine to treat advanced ovarian cancer. (Behavioral) | Trial development; Outreach; Enrollment/consent; Trial Engagement; Outcomes Assessment |
| Atherton 2007, UK  DOI: 10.1186/1745-6215-8-41 | Descriptive | Sexually Transmitted Infections [infectious diseases] | Sexually active female students under 28 years old; Focus on multiple racial or ethnic groups* | Questionnaire on sexual health, self-administered vaginal swabs and treatment if positive for chlamydia, as well as contact 1 month post sample collection to garner participants' understanding of when their samples would be tested. A follow up questionnaire assessed incidence of PID one year after participation. (Invasive) | Outreach; Trial Engagement |
| Bachour 2017, US  DOI: 10.1177/1740774516663461 | Comparative | Provoked vulvodynia [gynecologic conditions] | Black, Hispanic/Latino, and white women over 18 years old | Gabapentin vs. placebo (Pharmacologic) | Outreach; Enrollment/consent; Analysis & Interpretation |
| Bailey 2004, US,  DOI: 10.1016/j.apnr.2003.12.002 | Descriptive | Cervical cancer [sex-specific cancer] | Women > 14 years old; No racial or ethnic population focus | Women with cervical intra-epithelial neoplasia II or III postponed ablative therapy to receive topical reinoic acid treatment vs placebo, seen for 5 consecutive days. Follow up colposcopy after 12 weeks. Treated with ablative therapy for any remaining disease. (Invasive) | Outreach; Trial Engagement |
| Barnett 2012, US  DOI: 10.1016/j.cct.2012.06.005 | Primary qualitative | Breastfeeding [peripartum] | Low-income women >18 years old; Focus on multiple racial or ethnic groups | Two RCTs implemented interventions to increase breastfeeding quantity and duration. Trials compared lactation consultant-only, electronic prompt-only, and combined lactation consultant and electronic prompt interventions. (Behavioral) | Outreach; Trial Engagement; Outcomes Assessment |
| Barrera 2014, Other: worldwide  DOI: 10.2196/jmir.2999 | Descriptive | Postpartum depression [peripartum] | Pregnant women between 18 and 60 years old; No racial or ethnic population focus | Internet-delivered intervention to prevent postpartum depression. Study compared key words used to access a fully-automated website focused on post-partum depression and the association of key words with enrollment and consent in the study. (Behavioral) | Outreach; Enrollment/consent |
| Benham 2021, Canada  DOI: 10.2196/25208 | Comparative | PCOS [gynecologic conditions] | Women ages 18-40; No racial or ethnic population focus. | An RCT compared the effects of high-intensity interval training versus continuous aerobic exercise training on various health markers of women with Polycystic Ovarian Syndrome. (Behavioral) | Outreach |
| Benoit-Piau 2020, Canada  DOI: 10.1016/j.jsxm.2020.04.005 | Comparative | Vestibulodynia [gynecologic conditions] | Women ages 18-45; No racial or ethnic population focus | Large RCT compares topical lidocaine and PT to treat Vestibulodynia. (Pharmacologic) | Trial Development; Outreach; Enrollment/consent |
| Bleidorn 2015, Other: Germany  DOI: 10.3205/000221 | Primary qualitative | Urinary tract infection [infectious diseases] | NR; No racial or ethnic population focus | Treatment with fosfomycin vs ibuprofen and conditional antibiotic treatment if necessary. (Pharmacologic) | Trial Engagement |
| Blödt 2016, Other: Germany  DOI: 10.1136/bmjopen-2016-012592 | Primary qualitative | Menstrual pain [gynecologic conditions] | Women up to 34years old; No racial or ethnic population focus | Self-acupressure supported by smart phone 1-2 times per day x 6 min sessions 5 days before cycle and up to 6 times a day during pain days. (Other) | Outreach; Trial Engagement |
| Blumenthal 1995, US    PMID: 7721592 | Descriptive | Breast and cervical cancer screening [sex-specific cancer] | Women over > 18 years old; Black; low-income, inner-city community | Intervention included several sessions that included with discussion on breast and cervical cancer and screening tests (Pap smears, clinical breast exams, breast self-exams, and mammography). Six months post-intervention participants were interviewed again. (Behavioral) | Trial Development; Outreach; Enrollment/consent; Trial Engagement |
| Bonilla 2022, US  DOI: 10.1186/s13063-021-05945-8 | Descriptive | Breast cancer survivors [sex-specific cancer] | Women ages 30-65; Hispanic or Latino | Two studies tested 8-10 week cognitive-behavioral stress management programs with delayed control groups. (Behavioral) | Trial Development; Outreach; Study Engagement |
| Breitkopf 2011, US  DOI: 10.1525/jer.2011.6.3.31 | Primary qualitative | Non-invasive vaginal imaging [gynecologic conditions] | Women between 18-45 years; No racial or ethnic population focus | Testing the use of non-invasive method of vaginal imaging; (Invasive; Pharmacologic; Behavioral) | Trial Engagement |
| Brewster 2002, US  DOI: 10.1006/gyno.2002.6592 | Comparative | Abnormal Pap smear follow-up [sex-specific cancer] | Women over 18 years old; Hispanic/Latino | Randomized to usual care for Papsmear (with subsequent follow-up visit if abnormal) vs. single-visit program where women wait at clinic for same-day Pap smear results. (Invasive; Behavioral) | Outreach; Enrollment/consent |
| Brown 2002, US  DOI: 10.1016/s1049-3867(01)00145-1 | Primary qualitative | not specified | NR; No racial or ethnic population focus | Not specified. (Other) | Outreach; Trial Engagement |
| Brown 2012, US  DOI: 10.1016/j.cct.2012.03.003 | Comparative | Weight management [metabolic health] | Women ages 21-75; Focus on multiple racial /ethnic groups | Weight management trial. (Behavioral) | Outreach |
| Brown 2015, US  DOI: 10.1177/1740774514568125 | Comparative | Diabetes prevention postpartum [peripartum] | Pregnant women ages 20-45; No racial or ethnic population focus | Targeted recruitment letters that either included information about diabetes health risk targeted to race or ethnicity or did not. It was nested within a larger RCT examining the effect of lifestyle changes on pregnant women with gestational diabetes or impaired glucose tolerance.(Behavioral) | Outreach |
| Brubaker 2013, US  DOI: 10.1007/s00192-012-1824-x | Primary qualitative | Pelvic floor disorder [gynecologic conditions] | NR; No racial or ethnic population focus | Not specified. (Other) | Outreach |
| Burns 2008, US  DOI: 10.1177/0145721708325764 | Descriptive | Type 2 Diabetes [metabolic health] | Women over 55 years old; Black; rural population | Culturally sensitive, symptom-focused, disease management intervention to improve diabetes symptom knowledge, self-care practices, and health outcomes. Two intervention groups with intervention visits and one with a booster, was compared to an attentional control group. (Behavioral) | Trial Development; Outreach; Enrollment/consent; Trial Engagement |
| Butt 2010, Canada  DOI: 10.1016/j.cct.2010.06.003 | Comparative | Treatment for hotflashes [menopause] | Women ages 45-64 years old; No racial or ethnic population focus | Women were randomized to placebo vs. 300 mg of gabapentin for 6 weeks--4 weeks of treatment and a 2 week wean period. (Pharmacologic) | Outreach |
| Cambron 2001, US  DOI: [10.1067/mmt.2001.112567](https://doi.org/10.1067/mmt.2001.112567) | Comparative | Primary dysmenorrhea [gynecologic conditions] | Women ages 18-45; No racial or ethnic population focus | Low-back spinal manipulative therapy vs. placebo to decrease symptoms of dysmennorhea. (Other) | Outreach |
| Cantrell 2012, US  DOI: 10.1188/12.ONF.483-490 | Comparative | Childhood cancer survivorship [cancer survivorship] | Women ages 18-25; No racial or ethnic population focus | 8 sessions of Hope Intervention Program (HIP) specific strategies in small group interactive group format for cancer survivors. Control group got presentation on healthy lifestyle issues. (Behavioral) | Outreach; Trial Engagement |
| Carpenter 2016, UK  DOI: 10.3109/01443615.2015.1049988 | Descriptive | Cardiovascular function during pregnancy [peripartum] | Women over 18; No racial or ethnic population focus | Weekly supervised exercise program (land or water-based) starting at 20 weeks until full-term or could no longer participate in physical activity. Control arm was usual care. (Behavioral) | Outreach; Enrollment/consent |
| Catherine 2020, Canada  DOI: 10.1186/s13063-020-04328-9 | Descriptive | Child development education [peripartum] | Women ages 14-24; No racial or ethnic population focus | Women were randomized to receive NFP (a home-based nursing education intervention during pregnancy and postpartum) vs. existing prenatal/postnatal services. Intervention started perinatally and continued until children reach 2 years of age. (Behavioral) | Trial development; Outreach; Enrollment/consent; Trial Engagement; Outcomes Assessment |
| Choi 2016, US  DOI: 10.1097/FCH.0000000000000089 | Descriptive | Mammography and pap tests [sex-specific cancer] | NR; Asian | Twenty-nine Community Health Workers (CHWs) were used to recruit eligible Korean American women. The intervention consisted of 2-hour health literacy education, monthly phone counseling and navigation assistance for 6 months. At the end of the study, they conducted focus groups for the CHWs. (Behavioral) | Outreach; Trial Engagement |
| Cockayne 2005, UK  DOI: 10.1186/1471-2288-5-34 | Descriptive | Fracture prevention [bone health] | Community dwelling women over 70 years old; No racial or ethnic population focus | Parent trial tested calcium and vitamin D supplementation for fracture prevention. For this study, they tested whether offering study results in a follow up questionnaire. (Pharmacologic) | Outreach; Trial Engagement; Analysis & Interpretation; Dissemination |
| Coleman-Phox 2013, US  DOI: 10.5888/pcd10.120096 | Descriptive | Healthy pregnancy weight gain and stress reduction [peripartum] | Overweight and obese pregnant women ages 18-45; No racial or ethnic population focus | Phase 1: Focus groups to assess interest and identify barriers to intervention participation. Phase 2: Compared two group interventions that aimed to reduce stress-induced nonhomeostatic eating during pregnancy. (Behavioral) | Outreach; Trial Engagement |
| Daley 2007, UK  DOI: 10.1016/j.cct.2007.02.009 | Comparative | Exercise and breast cancer [sex-specific cancer] | Women ages 18-65 who had been treated for breast cancer; No racial or ethnic population focus | 8-week supervised exercise therapy intervention versus usual care, or an "equal contact exercise-placebo". (Behavioral) | Outreach; Enrollment/consent; Trial Engagement; Analysis & Interpretation |
| Daniels 2012, Australia  DOI: 10.1186/1479-5868-9-129 | Descriptive | Protective infant feeding practices [peripartum] | First time mothers over 18 years old; No racial or ethnic population focus | Comprehensive skills-based program using cognitive behavioral approach focused on feeding and parenting practices to mediate children's early feeding. Compared to control group where participants had self-directed access to typical child health services. (Behavioral) | Outreach; Enrollment/consent; Trial Engagement; Outcomes Assessment |
| DeBar 2009, US  DOI: 10.1016/j.cct.2009.02.007 | Comparative | Eating disorders [mental health] | Women ages 25-50 with eating disorders; No racial or ethnic population focus | Trial interventions were not described beyond "cognitive behavioral therapy-based guided self help program", as well as the different standard screening tools used to determine the criteria for the study and presence and severity of different aspects of binge eating. (Behavioral) | Outreach; Trial Engagement |
| Derose 2000, US  DOI: 10.1177/109019810002700508 | Comparative | Mammogram screening [sex-specific cancer] | Women ages 50-80; Black and Hispanic/Latino | Compared mail and telephone counseling to facilitate mammogram screening. (Behavioral) | Trial Development; Outreach; Enrollment/consent; Trial Engagement; Analysis & Interpretation |
| Dickson 2013, UK  DOI: 10.1186/1745-6215-14-389 | Descriptive | Pelvic organ prolapse [gynecologic conditions] | Women with pelvic organ prolapse; No racial or ethnic population focus | Individualized pelvic floor muscle training compared to an active control in which participants received a lifestyle advice leaflet. Invasive; (Behavioral) | Trial development; Outreach; Enrollment/consent; Trial Engagement; Analysis & Interpretation |
| Ekambareshwar 2018, Australia  DOI: 10.1186/s13063-018-2871-5 | Descriptive | Infant and childhood obesity [peripartum] | Pregnant women over 18 years old; No racial or ethnic population focus | Low intensity health promotion program with delivery of health behavior change messages via phone or text message. Data between were analyzed between those who consented and did not consent. (Behavioral) | Trial Development; Outreach; Trial Engagement |
| El-Khorazaty 2007, US  DOI: 10.1186/1471-2458-7-233 | Descriptive | smoking, depression, and IPV during pregnancy [peripartum] | Pregnant women over 18 and less than 28 weeks gestation; multiple racial/ethnic groups | Intervention group met with trained PAs (in addition to their physician) either immediately before or after a routine prenatal visit and at two postpartum sessions during which they received individualized counseling targeting their area(s) of risk. (Behavioral) | Outreach; Trial Engagement |
| Falcon 2011, US; Canada; Other: Puerto Rico  DOI: 10.1089/jwh.2010.2504 | Descriptive | HIV [infectious disease] | Women with HIV/AIDS; multiple racial/ethnic groups | 48 weeks of darunavir/ritonavir (DRV/r) 600 mg/100 mg twice daily combined with an investigator-selected optimized background regimen in ARV-experienced women and men. Control was not described. (Pharmacologic) | Trial Development; Outreach; Trial Engagement |
| Fitch 2020, US; Canada; Other: South Africa, Thailand, Botswana, Uganda, Zimbabwe, India, Haiti, Brazil, Peru  DOI: 10.1080/25787489.2020.1733794 | Comparative | Cardiovascular disease and HIV [infectious disease] | Men and women with HIV; multiple racial/ethnic groups | Pitavastatin calcium 4mg/day, length of intervention not specified. Control group received a placebo. (Pharmacologic) | Trial Development; Outreach; Enrollment/consent; Trial Engagement |
| Folmar 2001, US  DOI: 10.1016/s0197-2456(00)00117-3 | Comparative | Coronary atherosclerosis in postmenopausal women [menopause] | Women over age 55; multiple racial/ethnic groups | Estrogen replacement therapy with or without continuous low-dose progestin vs. placebo. (Pharmacologic) | Trial Development; Outreach; Enrollment/consent |
| Fouad 2004, US  DOI: 10.1016/j.cct.2004.03.005 | Comparative and descriptive | Causes of death and disability in postmenopausal women [menopause] | Postmenopausal women ages 50-79; multiple racial/ethnic groups | Dietary Modification (DM) arm examined the effects of a low-fat diet on prevention of cancer and heart disease; the Hormone-Replacement Therapy (HRT) component assesses the benefits and risks of HRT; and the Calcium and Vitamin D(CAD) component assesses the effects of the supplements in preventing osteoporosis and colon cancer. (Pharmacologic; Behavioral) | Outreach; Enrollment/consent; Trial Engagement; Analysis & Interpretation |
| Fouad 2014, US  DOI: 10.1002/cncr.28572 | Comparative | Low-grade cervical cytologic abnormalities [sex-specific cancer] | Low income women with low-grade cytologic abnormalities; Black | Community health advisors (CHAs) recruited and retained participants in a large RCT that evaluated optimal management of cytologic abnormalities. These trial participants were compared to those who received the standard protocol. (Behavioral; Invasive) | Trial Development; Outreach; Trial Engagement |
| Gerace 1995, US  DOI: 10.1016/s0197-2456(95)00041-0 | Comparative | Diet and nutrition [nutrition] | Women ages 50-79 and 50-65 (2 cohorts); No racial or ethnic population focus | Parent trial is a prevention trial. Trial described here is requesting a return mailing. Mailed recruitment letters (of different lengths) were distributed to participants. A second recruitment letter was mailed to each non-respondent 4 or more weeks after initial letter mailing. (Behavioral) | Trial Development; Outreach |
| Germino 2011, US  DOI: 10.1007/s11764-010-0150-x | Comparative and descriptive | Breast cancer [sex-specific cancer] | Women over 50 years old; Black | Psycho-educational intervention designed to help patients manage anxiety around cancer recurrence versus a control condition in which participants just discussed their experiences. (Behavioral) | Trial Development; Outreach; Enrollment/consent; Trial Engagement |
| Gillan 2009, UK  DOI: 10.1258/jms.2009.009023 | Comparative | Breast cancer screening [sex-specific cancer] | Women between ages 50-70; No racial or ethnic population focus | Patients receiving mammograms were randomly assigned to one of 3 protocols for reading of results (double reading using computer-aid; double reading only; single reading). (Other) | Trial Development; Outreach; Enrollment/consent |
| Goode 2008, US  DOI: 10.1016/j.jamcollsurg.2008.03.012 | Comparative | Pelvic organ prolapse [gynecologic conditions] | CARE study = women up to 70 years old; Colpocleisis study= women up to 80 years old; No racial or ethnic population focus | CARE study--participants were followed for 2 years after surgery; Colpocleisis study, participants were followed for 1 year after surgery. (Invasive) | Outreach; Enrollment/consent; Trial Engagement |
| Goodwin 2000, Canada  DOI: 10.1016/s0895-4356(99)00148-1 | Descriptive | Metastatic breast cancer [sex-specific cancer] | NR; No racial or ethnic population focus | Weekly, 90-min therapist-led support group that adhered to the principles of expressive-supportive therapy compared to usual care plus educational materials. (Behavioral) | Outreach; Trial Engagement |
| Griffin 2013, Australia  DOI: 10.6133/apjcn.2013.22.2.16 | Comparative and descriptive | Overweight and obesity [metabolic health] | Women ages 18-25; No racial or ethnic population focus | The study compared two diets, both arms involving regular face to face visits for dietetic counselling and behavior modification therapy and followed a standardized exercise program (30 min of brisk walking daily). (Behavioral) | Outreach; Trial Engagement |
| Heard 2012, US  DOI: 10.1111/j.1741-6612.2011.00573.x | Comparative | Osteoporotic fracture [bone health] | Women ages 65 - 80; No racial or ethnic population focus | A "medication" arm (intervention) for 2-months was compared to a placebo in phase III trial to evaluate safety and efficacy. (Pharmacologic) | Outreach; Analysis & Interpretation |
| Heiney 2010, US  DOI: 10.1188/10.ONF.E160-E167 | Comparative and descriptive | breast cancer [sex-specific cancer] | Women over 21 years old; Black | 8 weekly teleconference "therapeutic group" sessions followed by two booster sessions that were two weeks apart vs. usual care. (Behavioral) | Trial development; Outreach; Trial Engagement |
| Hemminki 2004, Estonia  DOI: 10.1016/j.jclinepi.2004.04.009 | Descriptive | menopause [menopause] | Postmenopausal women ages 50-64; No racial or ethnic population focus | A primary prevention trial with postmenopausal hormone therapy (PHT). Women received PHT, placebo or nothing, and some were blinded to treatment, and some were not. (Pharmacologic) | Outreach; Enrollment/consent |
| Homer 2000, Australia  DOI: 10.1054/midw.2000.0230 | Descriptive | maternity care models [peripartum] | Pregnant women (less than 24 weeks gestation); No racial or ethnic population focus | The STOMP trial compared a community-based continuity of midwifery care model with standard maternity hospital care in a culturally diverse area. (Other) | Trial Development; Outreach; Enrollment/consent; Trial Engagement |
| Houghton 2018, UK  DOI: 10.1111/1471-0528.15333 | Primary qualitative | postpartum hemorrhage [peripartum] | Women who had declined participation in a larger clinical trial focused on maternal health; No racial or ethnic population focus | Clinical trial examined administration of tranexamic acid vs placebo to reduce risk of death. In-depth qualitative interviews were conducted about the acceptability of recruitment methods. (Pharmacologic) | Enrollment/consent |
| Irwin 2008, US  DOI: 10.1002/cncr.23446 | Comparative | breast cancer survival [sex-specific cancer] | Post-menopausal breast cancer survivors ages 40 to 75 years old; No racial or ethnic population focus | Participants were randomly assigned to exercise or usual care groups. (Behavioral) | Trial Development; Outreach; Trial Engagement |
| Ivaz 2006, UK  DOI: 10.1093/fampra/cmi109 | Descriptive | Chlamydia and PID [infectious disease] | Women younger than 27 years old; No racial or ethnic population focus | Participants completed a sexual health questionnaire, self-administered vaginal swab, and then a follow-up questionnaire after 1 year. Half of the swabs are randomized to be tested for chlamydia and half are stored for a year. (Other) | Outreach; Trial Engagement |
| Johnson 2015, US  DOI: 10.1111/cts.12264 | Descriptive | Metabolic syndrome [metabolic health] | Women with metabolic syndrome; Black | Intervention group received drug for insulin sensitivity vs. placebo with seven follow-up clinic visits. (Pharmacologic) | Outreach |
| Jones 2017, US  DOI: 10.1007/s10461-017-1797-3 | Comparative | HIV prevention [infectious disease] | Women ages 18-29 who had sexual relationships with men within the last 3 months; Black | Participants viewed a series of soap operas with a video commentator to reduce HIV risk and increase HIV testing. Control was a series of episodes of a romantic comedy and 1 episode of a CDC-based HIV prevention video. (Behavioral) | Trial Development; Outreach; Enrollment/consent; Trial Engagement |
| Kenyon 2006, worldwide  DOI: 10.1136/qshc.2005.015636 | Primary qualitative | antibiotics for pre-term labor [peripartum] | Participants in the ORACLE trial; No racial or ethnic population focus | ORACLE trial: 2x2 factorial design; augmentin/not and erythromycin/not; 4 times daily x 10 days or until delivery. (Pharmacologic) | Dissemination |
| Kozica 2015, Australia  DOI: 10.1186/s13063-015-0860-5 | Primary qualitative | prevention of weight gain [metabolic health] | Women ages 18-55 years old; No racial or ethnic population focus | Evidence-based healthy lifestyle weight gain prevention. (Behavioral) | Trial Development; Outreach; Trial Engagement; Outcomes Assessment |
| Koziol-McLain 2016, New Zealand  DOI: 10.2196/jmir.6515 | Comparative | interpersonal violence [IPV] | Women over 16 years old experiencing IPV; No racial or ethnic population focus | Web-based safety decision aid for women experiencing IPV in current relationship was tested. (Behavioral) | Trial Development; Outreach; Enrollment/consent; Trial Engagement |
| Larkey 2002, US  DOI: 10.1016/s0197-2456(02)00190-3 | Descriptive | multiple: cardiovascular disease, cancer and osteoporosis prevention [general women's health] | Women ages 50-74 years old; Hispanic/Latino | The study tested the effectiveness of training WHI participants to become lay advocates for the program (Embajadoras, or Hispanic Ambassadors) and to compare them to a group of similar women who were not trained but who received WHI brochures to share. (Other) | Outreach; Enrollment/consent; Trial Engagement; Analysis & Interpretation |
| Le 2008, US, Mexico  DOI: 10.1007/s00737-008-0009-6 | Comparative between | postpartum depression [peripartum] | Pregnant women ages 18-35 years old; Hispanic/Latino | 8-week antenatal psycho-educational group intervention to prevent postpartum depression. Compared to usual care. (Behavioral) | Trial Development; Outreach |
| Leavitt 2017, US  DOI: 10.1007/s13142-016-0450-4 | Comparative | smoking and pregnancy [peripartum] | Pregnant female smokers over 14 years old; No racial or ethnic population focus | The use of text messages to recruit participants (and then to quit smoking). Control group received messages unrelated to smoking cessation. (Behavioral) | Outreach |
| Leonard 2014, Australia  DOI: 10.1186/1745-6215-15-23 | Comparative | Iron supplementation; web-based food diary; weight management [nutrition] | Women were aged 18-35 years old in one trial and 18-30 years old in the other two trials; No racial or ethnic population focus | Three different trials were focused on dietary interventions and weight management. One focused on iron supplementation in iron deficient women and effect on cognitive function. One looked at the use of web-based food diaries vs. paper-based diaries vs. smartphone-based diaries. And one was an on online survey about expectations of an online weight management program. (Pharmacologic; Behavioral) | Outreach; Enrollment/consent; Trial Engagement |
| Lesher 2015, US  DOI: 10.1111/ppe.12177 | Comparative | Effects of aspirin in gestation and reproduction [peripartum] | Women 18-40 years old who had suffered 1-2 previous pregnancy losses and were trying to conceive; No racial or ethnic population focus | The Effects of Aspirin in Gestation and Reproduction (EAGeR) trials not well described in this paper, but it required multiple biospecimen (blood, urine) collections and study visits through preconception and pregnancy. (Pharmacologic) | Outreach; Enrollment/consent |
| Lindenstruth 2006, US  PMID: 17061750 | Comparative | Effects of soy on lipid profile [menopause] | Postmenopausal women under age 79; Black | Participants were randomized to soy vs. placebo daily supplement for 12 weeks with lipid profile checked. (Pharmacologic) | Outreach |
| Lopez 2008, US  DOI: 10.1080/14622200701704962 | Comparative | Smoking cessation (prevention of relapse) [peripartum] | Women who were ex-smokers and either pregnant or post-partum and over 18 years old; No racial or ethnic population focus | Women randomized to use of a smoking abstinence education booklet ("Forever Free for Baby and Me") vs. usual care. Outcome was smoking abstinence at 12-months postpartum. (Behavioral) | Outreach; Enrollment/consent; Trial Engagement |
| MacLachlan 2021, UK  DOI: 10.1186/s13063-021-05348-9 | Comparative | Peripartum [peripartum] | Women over 14 years old; No racial or ethnic population focus | Pregnant women were randomized into 2 different group-based parenting interventions on maternal mental health and mother-child interactions. (Behavioral) | Outreach; Enrollment/consent; Intervention delivery; Outcome Assessment |
| Martin 2013, US  DOI: 10.1353/hpu.2013.0125 | Descriptive | Postpartum depression prevention [peripartum] | English or Spanish speaking, at least 18 years old, and had delivered a healthy infant; Black; Hispanic/Latino | Trial studied a behavioral education intervention and used in-hospital survey plus 3 additional surveys by phone over 6 months. (Behavioral) | Trial Development; Outreach; Trial Engagement |
| Maxwell 2005, US  DOI: 10.1007/s10900-004-1956-0 | Comparative | Breast and cervical cancer screening [sex-specific cancer] | Women over 40 years old; Asian | Filipino American women were randomized to participate in group sessions that were either a cancer screening module or a physical activity module. (Behavioral) | Trial Development; Outreach; Trial Engagement |
| McDermott 2004, US  DOI: 10.1002/eat.10231 | Comparative | anorexia nervosa and bulimia nervosa [mental health] | Women ages 14-50 who met the criteria for anorexia nervosa in the previous 2 years; No racial or ethnic population focus | One study compared CBT to CBT +Fluoxetine. Recruitment was compared with a bulimia study that was not as well described. The bulimia study did not specify the intervention (possibly the same as anorexia study). (Pharmacologic; Behavioral) | Outreach |
| Menon 2008, UK  DOI: 10.1136/bmj.a2079 | Comparative and descriptive | ovarian cancer screening [menopause] | Postmenopausal women ages 50-74 were recruited to the UK Collaborative Trial of Ovarian Cancer Screening; No racial or ethnic population focus | The multisite trial included 200,000 women randomized to annual screening with serum CA 125 or transvaginal ultrasound vs. no intervention. (Invasive; Other) | Trial Development; Outreach; Enrollment/consent; Trial Engagement |
| Messer 2006, US  DOI: 10.1007/s11255-006-0018-1 | Descriptive | urinary incontinence [urogynecology] | Continent, postmenopausal women ages 55-80 years old; No racial or ethnic population focus | Behavioral modification program to prevent the onset of UI among post-menopausal women. Women in the treatment arm were trained in behavioral techniques and followed for 12 months. Compared to wait-list control group. (Behavioral) | Outreach; Trial Engagement; Analysis & Interpretation |
| Moody 1995, US  DOI: 10.1111/j.1745-7599.1995.tb01116.x | Descriptive | Post-menopausal bone density loss [menopause] | Women 3 years post menopause; Black | Treatment arm received alendroate vs. control arm received standard treatment of calcium and vitamin D. (Pharmacologic) | Trial development; Outreach; Enrollment/consent; Trial Engagement; Analysis & Interpretation |
| Newlin 2006, US  PMID: 17061753 | Descriptive | Type 2 Diabetes Education Program [metabolic health] | Women ages 18-65 with Type 2 Diabetes; Black | The RCT (not described in this article): 11-week culturally relevant group diabetes self-management training (DSMT), coping skills training (CST), and diabetes care intervention was compared to a 10-week usual diabetes education and diabetes care intervention on physiological and psychosocial outcomes. (Behavioral) | Trial development; Outreach; Enrollment/consent; Trial Engagement; Analysis & Interpretation |
| Nicklas 2016, US  DOI: 10.1007/s10995-015-1825-8 | Descriptive | Gestational DM [peripartum] | Pregnant or postpartum women ages 18-45, who had had gestational diabetes; No racial or ethnic population focus | Participants were randomized into web-based Balance After Baby lifestyle intervention or control. (Behavioral) | Trial Development; Enrollment/consent; Trial Engagement; Analysis & Interpretation |
| Oakley 2003, UK  DOI: 10.1080/13557850303554 | Descriptive | Support for women with infants [peripartum] | NR; No racial or ethnic population focus | Participants participated in one of two alternative strategies for providing support to women with infants: 1) a program of home visits from research health visitors training in supportive listening, or 2) support provided from local community groups. (Behavioral) | Outreach; Enrollment/consent |
| Ott 2006, US  DOI: 10.1097/00002820-200601000-00004 | Comparative and descriptive | Osteoporosis in breast cancer survivors [sex-specific cancer] | Women ages 35-75; No racial or ethnic population focus | Women were randomized to risedronate+Ca/Vit D supplementation only or risedronate+Ca/Vit D AND exercise routine. (Pharmacologic; Behavioral) | Outreach; Enrollment/consent; Trial Engagement; Outcomes Assessment |
| Paine 2008, UK; Australia; Other: New Zealand  DOI: 10.1186/1745-6215-9-5 | Comparative | Menopause [menopause] | Women ages 50-69 years of age; No racial or ethnic population focus | 10 years of estrogen therapy; 10 years of estrogen + progestogen therapy or placebo; 5 years of follow up after treatment. [RCT is not described in depth]. (Pharmacologic) | Outreach |
| Panjari 2008, Australia  DOI: 10.1089/jwh.2007.0732 | Descriptive | Postmenopausal sexual function [menopause] | Menopausal, sexually active women ages 40-65; No racial or ethnic population focus | DHEA 50mg daily versus placebo. (Pharmacologic) | Outreach; Enrollment/consent |
| Paquin 2021, US  DOI: 10.1111/cts.12950 | Comparative | Fragile X syndrome (newborns) [peripartum] | Mothers of newborns; No racial or ethnic population focus | Researchers are evaluating direct mail and email outreach approaches implemented during phase 1 of a multi-phase recruitment strategy for "Early Check", the parent study. (Other) | Outreach; Trial Engagement |
| Park 2007, US  DOI: 10.1016/j.ypmed.2006.10.008 | Comparative | Smoking cessation during pregnancy [peripartum] | Pregnant smokers over 18 years old; No racial or ethnic population focus | Testing the efficacy of a telephone counseling program for pregnant smokers. Control group received a 5-min advice to quit call and a self-help booklet. (Behavioral) | Enrollment/consent; Trial Engagement |
| Pastore 2009, US  DOI: 10.1016/j.ctim.2009.03.004 | Descriptive | Polycystic ovary syndrome [general women's health] | Women between the ages of 18 and 43, with a diagnosis of PCOS; No racial or ethnic population focus | The study used 5-month protocol: a 2-month intervention (1:1 randomization of real:sham acupuncture) and 3 months of follow-up. (Invasive; Other) | Trial Development; Outreach; Trial Engagement |
| Peindl 2003, US  DOI: 10.1016/s0022-3956(02)00086-9 | Comparative and descriptive | Postpartum depression [peripartum] | Women who were either pregnant or post-partum; No racial or ethnic population focus | Two trials described: 1) A randomized, double-blind, clinical trial to determine the efficacy of nortriptyline compared to placebo for the prevention of postpartum depression. 2) A randomized, double-blind, controlled clinical trial comparing nortriptyline versus sertraline for the treatment of postpartum depression. (Pharmacologic; Behavioral) | Outreach; Enrollment/consent |
| Phillippi 2018, US  DOI: 10.1016/j.jogn.2018.04.134 | Descriptive | Prenatal care for moderate-risk women [peripartum] | NR; No racial or ethnic population focus | A new format of midwife-physician collaboration for prenatal care of moderate-risk women. (Other) | Outreach; Enrollment/consent |
| Phipps 2013, Australia  DOI: 10.1111/aogs.12243 | Comparative | Occipitoposterior (OP) positioning of fetus at the start of labor [peripartum] | Pregnant women over 18 years old; No racial or ethnic population focus | Women with occipitoposterior positioning in labor (confirmed by ultrasound) were randomized to manual rotation or 'sham' vaginal examination. (Invasive) | Outreach; Enrollment/consent |
| Pinto 2021, US    DOI: 10.1016/j.cct.2021.106285 | Comparative | Breast cancer survivors [sex-specific cancer] | Women breast cancer survivors >21 years old; No racial or ethnic population focus | Breast cancer survivors were randomized into one of three intervention groups promoting physical activity (3 months) and various forms of maintenance support (6 months). (Behavioral) | Trial Development; Outreach; Enrollment/consent; Trial Engagement; Outcomes Assessment |
| Pollak 2006, US  DOI: 10.1080/14622200600789882 | Comparative and descriptive | Smoking cessation during pregnancy [peripartum] | Pregnant women over 18 years old who reported smoking >5 cigarettes / day during pregnancy; No racial or ethnic population focus | Participants were randomized to use of nicotine replacement (patch, lozenge, gum). They completed surveys (2 in pregnancy, 1 postpartum) and saliva samples to assess their smoking status. Compared to usual care. (Pharmacologic) | Trial Development; Outreach; Trial Engagement |
| Pribulick 2010, US,  PMID: 23641192 | Descriptive | CVD risk reduction [cardiovascular disease] | Women between the ages of 55 and 65 in two rural communities; Black | Half received nurse intervention (4 visits) before community intervention and half received nurse visits after community intervention. (Behavioral) | Trial Development; Outreach; Trial Engagement |
| Price 2019, Australia  DOI: 10.1186/s12913-019-4698-5 | Comparative and descriptive | Childhood development [peripartum] | Pregnant women; No racial or ethnic population focus | Intervention included 25 home visits by nurse between pregnancy and 2 years old to provide education about parenting, home learning vs. standard of care. Compared to usual care. (Behavioral) | Trial Development; Outreach; Enrollment/consent; Trial Engagement; Outcomes Assessment |
| Ramos-Gomez 2008, US  DOI: 10.1177/1740774508093980 | Comparative and descriptive | Early childhood dental caries [peripartum] | Women ages 18-33; Hispanic/Latino | Women and infants were randomized to oral health counseling vs. couseling+chlorhexidine rinse and fluoride varnish. (Pharmacologic) | Trial Development; Outreach; Enrollment/consent; Trial Engagement |
| Rdesinski 2008, US  DOI: 10.1353/hpu.0.0016 | Comparative and descriptive | contraception through home visits [contraception] | Women ages 15-44 and at risk for pregnancy and with low socioeconomic indicators; No racial or ethnic population focus | An RCT examining nurse-delivered contraceptives in the home. The intervention groups received family planning counseling and a 3-month supply of hormonal contraception. Control group received family planning counseling and no contraception. (Pharmacologic) | Outreach; Enrollment/consent; Trial Engagement; Outcomes Assessment |
| Russell 2008, US  DOI: 10.1016/j.apnr.2006.05.001 | Primary qualitative | breast cancer screening [sex-specific cancer] | Women ages 41-75; Black | Interactive computer-assisted instruction program vs culturally appropriate video, culturally appropriate pamphlet. (Behavioral) | Outreach; Trial Engagement |
| Salihu 2015, US  PMID: 27621990 | Descriptive | Use of folic acid in pregnant women who smoke. [peripartum] | Women who were less than 20 weeks pregnant; No racial or ethnic population focus | Women at <20 wks gestation were screened for smoking. Both treatment and control groups were enrolled in smoking cessation programs. Non-smokers were also enrolled and assessed until baseline. Control group received .8mg daily folic acid. Treatment group received 4.0 mg/day folic acid. Pharmacologic | Trial Development; Enrollment/consent; Trial Engagement |
| Sanders 2009, Australia  DOI: 10.1186/1471-2288-9-78 | Comparative | fracture risk [bone health] | Women over 70 years old and at increased risk of fracture; No racial or ethnic population focus | Participants in the treatment group receive 500,000 IU vit D3 every autumn for 5 consecutive years compared to placebo. (Pharmacologic) | Outreach; Analysis & Interpretation |
| Santoyo Olsson 2019, US  DOI: 10.1371/journal.pone.0224068 | Descriptive | Breast cancer survivors [sex-specific cancer] | Women (age NR); Hispanic or Latino | Breast cancer survivors were randomized to a 10-week intervention in which peer educators taught cognitive-behavioral coping skills or to a wait-list control group. (Behavioral) | Trial Development; Outreach; Trial Engagement; Outcomes Assessment |
| Sharp 2008, US  DOI: 10.1123/jpah.5.6.870 | Comparative and descriptive | Obesity [metabolic health] | Women ages 30-65 with a BMI between 30 kg/m2 and 50 kg/m2; Black | Participants met in small group sessions that included didactics, weigh in, exercise, and discussion. They were also offered weekend opportunities to exercise with a group and had target step goals. Control group received a general health newsletter once a week. (Behavioral) | Trial Development; Outreach |
| Shere 2014, Canada  DOI: 10.1371/journal.pone.0092744 | Comparative | Folic acid supplementation during pregnancy [peripartum] | Women ages 18-45, were pregnant or trying to conceive and not taking folic acid supplements; No racial or ethnic population focus | Participants were assigned prenatal vitamins containing either 1.5 or 5 mg folic acid. They have 4 blood draws during their pregnancy (baseline, 6 wks, 12 wks, and 30 wks gestation). (Pharmacologic) | Outreach |
| Shuhatovich 2005, US  DOI: 10.1016/j.ygyno.2005.07.093 | Comparative | Cervical cancer screening [sex-specific cancer] | Women who were not pregnant and were at least 18 years old; No racial or ethnic population focus | Study is testing a new optical technology, fluorescence and reflectance spectroscopy, for detecting cervical dysplasia. Participants undergo Pap smears and colposcopically directed biopsy, as well as optical spectroscopy. (Invasive) | Outreach |
| Smith 2006, Australia  DOI: 10.1016/j.ctim.2005.07.004 | Comparative | Complementary and alternative therapies for pregnancy related conditions [peripartum] | NR; No racial or ethnic population focus | This paper describes recruitment for 5 RCTs examining the use of acupuncture and herbal medicine during pregnancy. Two sought to reduce symptoms of morning sickness; one looked at stimulating the onset of labor; one looked at increasing pregnancy rates during embryo transfer during IVF treatment, and one looked at efficacy in expelling retained placenta. (Other) | Outreach; Trial Engagement |
| Smith 2007, US  DOI: 10.1177/1740774506075863 | Comparative | Dietary change interventions [nutrition] | Women over 40 years old; No racial or ethnic population focus | Trial comparing dietary change intervention delivery. Participants were recruited using two methods: letter plus phone call versus letter plus interactive recruitment website. (Behavioral) | Outreach; Enrollment/consent |
| Smyth 2009, UK  DOI: 10.1111/j.1523-536X.2009.00326.x | Descriptive | Pre-eclampsia [peripartum] | Women in the UK who had participated in the Magpie trial (international trial studying the use of magnesium sulfate for preeclampsia); No racial or ethnic population focus | MgSO4 for pregnant/postpartum people to reduce risk of preeclampsia versus placebo. (Pharmacologic) | Outreach; Enrollment/consent; Trial Engagement; Analysis & Interpretation |
| Staffileno 2006, US  DOI: 10.1097/00005082-200605000-00009 | Comparative | Hypertension and exercise [cardiovascular disease] | Women ages 18-45 at risk for hypertension; Black, | A home-based, physical activity trial consisting of lifestyle physical activity, physical activity logs, and wearing a portable heart rate monitor. The "no-exercise" group were instructed to continue with daily activities. (Behavioral) | Trial Development; Outreach; Trial Engagement |
| Stendell-Hollis 2011, US  DOI: 10.1016/j.cct.2011.03.007 | Comparative | Reducing anthropometric measures in lactating women. [peripartum] | Lactating women between 18 and 40 years old; No racial or ethnic population focus | Diet modification including nutrition education, lifestyle counselling, and support to adopt and adhere to assigned diet via education with dietitian vs. USDA's MyPyramid diet for Pregnancy and Breastfeeding. (Behavioral) | Trial Development; Outreach; Enrollment/consent; Trial Engagement |
| Stratton 2015, US  DOI: [10.1177/1740774514566333](https://doi.org/10.1177/1740774514566333) | Descriptive | HPV [sex-specific cancer] | Women with "recent Pap smear results with HSIL or where HSIL cannot be ruled out"; No racial or ethnic population focus | In a trial of HPV vaccine for therapeutic purposes, participants received one of four doses of new HPV vaccine. (Pharmacologic) | Outreach; Enrollment/consent; Trial Engagement |
| Sturgeon 2018, US  DOI: 10.1002/cncr.30935 | Comparative | Exercise and weight loss effects on lymphedema (BCRL) [sex-specific cancer] | Cancer survivors who were at least 6 months post-treatment; Black | Four groups of participants received 2 lymphedema compression garments and ongoing lymphedema care. The four arms included: no intervention; exercise program alone; weight loss program alone; combined exercise program and weight loss program. (Behavioral) | Trial Development; Outreach |
| Swaine 2011, US  DOI: 10.1111/j.1365-2788.2011.01399.x | Descriptive | Cervical and breast cancer screening [sex-specific cancer] | Women over 18 years old with intellectual disabilities; No racial or ethnic population focus | Women Be Healthy trial: Classroom-based intervention focused on educating about the importance of cervical and breast cancer screening, screening procedures, and symptom reporting. Wait-list control. (Behavioral) | Trial Development; Outreach; Enrollment/consent; Trial Engagement |
| Sweet 2008, US  DOI: 10.1016/j.cct.2007.11.003 | Comparative and descriptive | Unspecified reproductive health [general women's health] | Women ages 12-45; Black | Researchers describe recruitment methods for reproductive age Black women (ages 12-45) into research compared to parallel efforts recruiting Caucasian women at a different study site. The trial itself is not described. (Other) | Trial Development; Outreach; Trial Engagement |
| Tanjasiri 2015, US  DOI: 10.1353/cpr.2015.0067 | Descriptive | Cervical cancer screening [sex-specific cancer] | Women ages 21-65 in long-term heterosexual relationships; Native Hawaiian or Other Pacific Islander | A community trial promoting Pap testing through gender-specific social support-informed intervention sessions. Intervention was compared to a gender-specific "usual care" control session. (Invasive; Behavioral) | Trial development; Outreach; Enrollment/consent; Trial Engagement; Outcomes Assessment |
| Tworoger 2002, US  PMID: 11815403 | Comparative | Effect of moderate exercise on endogenous sex hormones [menopause] | Postmenopausal women ages 50-75; No racial or ethnic population focus | Year-long trial studying an exercise intervention on levels of endogenous hormones in postmenopausal women versus stretching-only exercise. (Behavioral) | Outreach; Analysis & Interpretation |
| Unson 2004, US  DOI: 10.1177/0898264304268588 | Comparative | Effect of estradiol on bone density [bone health] | Postmenopausal women over the age of 65; Black or Hispanic/Latina | (From original RCT): Postmenopausal women randomized to daily estradiol (+/- progesterone) vs. placebo on bone density measured every year x 3 years. (Pharmacologic) | Trial Development; Outreach; Trial Engagement; Outcomes Assessment |
| Usadi 2015, US  DOI: 10.1016/j.cct.2015.09.010 | Comparative | Pregnancy in PCOS; multiple intrauterine gestations from ovarian stimulation [peripartum] | Women ages 18-40 with PCOS; No racial or ethnic population focus | 1) (PPCOS II) 20-week trial of clomiphene citrate and letrozole for infertility in women with PCOS; 2) AMIGOS 3-armed trial of treatment of couples with unexplained fertility with letrozole vs clomiphene citrate vs gonadotropins on multiple gestations. (Pharmacologic) | Trial Development; Outreach; Analysis & Interpretation |
| Van der Worp 2020, Netherlands  DOI: 10.1016/j.jclinepi.2019.12.001 | Comparative | Urinary incontinence [urogynecology] | Women over 18 years old; No racial or ethnic population focus | From RCT article: Intervention group is randomized to use an online app for behavior training to treat UI. Control group received usual care from their GP, who discussed care options. (Behavioral) | Outreach |
| Velott 2008, US  DOI: 10.1016/j.whi.2008.02.002 | Comparative | Pre-and interconceptional risk reduction [peripartum] | Women ages 18-35; No racial or ethnic population focus | Behavioral intervention for pre- and interconceptional women to reduce modifiable risk factors. (Behavioral) | Trial Development; Outreach; Enrollment/consent; Trial Engagement |
| Vignato 2019, US  DOI: 10.1007/s00737-018-0894-2 | Comparative | Depression [peripartum] | Pregnant women 16 to 25 weeks gestation; No racial or ethnic population focus | Randomized women to individual vs. group online depression prevention program over 20 weeks. (Behavioral) | Outreach |
| Vogsen 2020, Denmark  DOI: [10.1186/s40900-019-0174-y](https://doi.org/10.1186/s40900-019-0174-y) | Comparative and descriptive | Advanced breast cancer [sex-specific cancer] | Women with metastatic breast cancer; No racial or ethnic population focus | Women were randomized to use of PET/CT vs. standard CT scan every 3 months for evaluation of response to metastatic breast cancer treatment. (Other) | Trial Development; Outreach; Enrollment/consent; Trial Engagement; Analysis & Interpretation; Dissemination |
| Vollert 2020, Germany  DOI: 10.1002/eat.23250 | Comparative | Anorexia nervosa prevention [mental health] | Women over 18 years old at high risk of eating disorders; No racial or ethnic population focus | Participants were randomized to receive a 10-week internet-based intervention about eating disorders. Intervention also included diaries, forums, and feedback by psychologist. Wait list control. (Behavioral) | Trial Development; Outreach; Enrollment/consent; Trial Engagement; Analysis & Interpretation |
| Waltman 2019, US  DOI: 10.1097/NNR.0000000000000356 | Comparative | Menopause and bone density. [menopause] | Women within 5 years of menopause with a diagnosis of osteopenia; No racial or ethnic population focus | Three intervention groups: Control group received 12 months of calcium and vitamin D supplements only; Intervention group that received supplements plus a bisphosphonate medication (risedronate group); 2^nd^ intervention group received supplements plus a three-time weekly structured bone-loading exercise program (exercise group). (Pharmacologic; Behavioral) | Trial Development; Outreach |
| Webb 2010, US  DOI: 10.1186/1471-2288-10-88 | Descriptive | Preterm delivery [peripartum] | Women who had previously delivered a child at less than 35 weeks gestation; No racial or ethnic population focus | Intervention group underwent screening and referral for further treatment for 6 risk factors (infection, periodontal disease, smoking, inadequate nutrition, depression, maternal stress) vs. usual care. (Behavioral) | Trial Development; Outreach; Trial Engagement; Outcomes Assessment; Analysis & Interpretation |
| Wiemann 2005, US  DOI: 10.1016/j.jpag.2005.09.006 | Descriptive | STI screening (chlamydia and gonorrhea infection) [infectious disease] | Women ages 16-21.5 years old; Black and Hispanic/Latino | Participants were randomized to a "behavioral intervention" over 12-months aimed to increase frequency of STI testing vs. standard of care. (Behavioral) | Trial Development; Outreach; Enrollment/consent; Trial Engagement |
| Wilbur 2013, US  DOI: 10.1002/nur.21550 | Comparative and descriptive | lifestyle physical activity to improve cardiovascular health in midlife AA women [cardiovascular disease] | Sedentary women ages 40-65; Black | All arms included a lifestyle physical activity prescription with an accelerometer for self-feedback and monitoring, as well as small group meetings targeted to increase lifestyle physical activity. Between visits, participants in the telephone contact condition received motivational telephone calls, and participants in the automated computer-linked telephone contact condition received motivational messages. The control group did not receive any telephone contact. (Behavioral) | Trial Development; Outreach; Trial Engagement |
| Wilbur 2001, US  DOI: 10.1097/00005082-200104000-00008 | Comparative | cardiovascular disease [cardiovascular disease] | Women ages 45-65 years old employed at least 20 hours/week; Black | Year-long home-based walking intervention (improving aerobic fitness, blood pressure, and lipoprotein levels). Wait list control. (Behavioral) | Trial Development; Outreach; Trial Engagement; Analysis & Interpretation |
| Zhu 2000, US  PMID: 10976173 | Descriptive | breast cancer screening [sex-specific cancer] | Women over 65 years old in one of five housing complexes; Black | Community-based intervention randomized 5 housing complexes to an educational intervention program on breast cancer screening, mental health over 3 years compared to controls (5 housing complexes without intervention). (Behavioral) | Trial Development; Outreach; Enrollment/consent; Trial Engagement |

*Multiple racial/ethnic groups: study either sought to recruit multiple groups exclusively or to over-recruit from multiple specific racial and ethnic groups.
