# Supplementary figures and images for: Strategies for enhancing the representation of women in clinical trials: an evidence map
Source: Syst Rev. 2024 Jan 2;13:2. doi: 10.1186/s13643-023-02408-w (PMC10759390; doi:10.1186/s13643-023-02408-w)

#
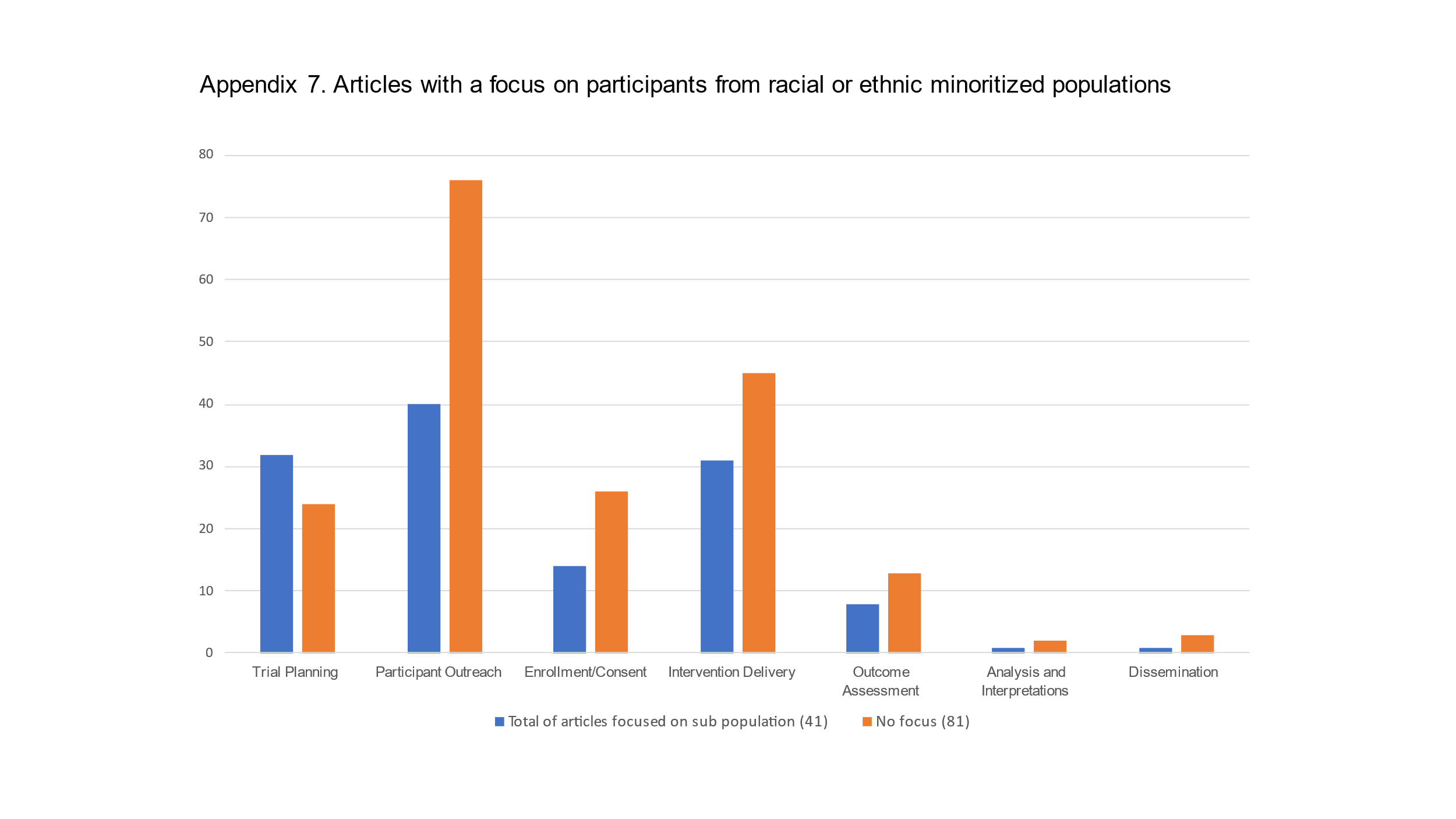

Supplement: Supplementary file 7 — Additional file 7: Appendix 7. Articles with a focus on participants from racial or ethnic minoritized populations. [file 13643_2023_2408_MOESM7_ESM.docx]

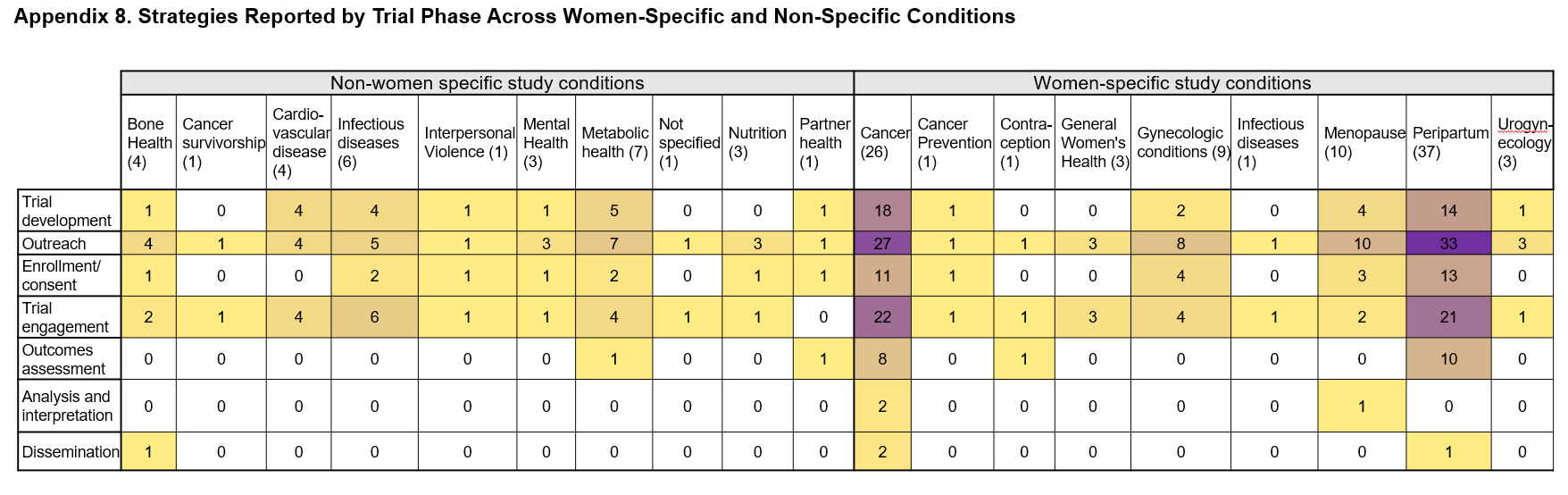

Supplement: Supplementary file 8 — Additional file 8: Appendix 8. Strategies reported by trial phase across women-specific and non-specific conditions. [file 13643_2023_2408_MOESM8_ESM.docx]
