# Supplementary material for: Strategies for enhancing the representation of women in clinical trials: an evidence map
Source: Syst Rev. 2024 Jan 2;13:2. doi: 10.1186/s13643-023-02408-w (PMC10759390; doi:10.1186/s13643-023-02408-w)
Supplement: Supplementary file 9 — Additional file 9: Appendix 9. Author-Reported Conclusions from Included Articles. [file 13643_2023_2408_MOESM9_ESM.docx]

# Appendix 9. Author-Reported Conclusions from Included Articles

| **Author, year** | **Conclusions as reported by included articles*** |
| --- | --- |
| **Non-women-specific study conditions** | |
| Bone health | |
| Cockayne 2005 | Offering study results to women living in the community aged over 70 does not increase response rates to postal questionnaires. Although researchers have an ethical obligation to offer participants study results, since 10% of women did not wish to receive the results, investigators should give participants the option to opt out of receiving the study's results. DOI: 10.1186/1471-2288-5-34 |
| Heard 2012 | Obtaining a specific study cohort can be achieved by a comprehensive, targeted, rapid recruitment program. A research center database search was the most successful and cost-effective recruitment modality in this small study.  DOI: 10.1111/j.1741-6612.2011.00573.x |
| Sanders 2009 | The most successful recruitment strategy was the targeted mail-out and the response rate was no higher in the local region where the study had extensive exposure through other recruiting strategies. The strategies that were labour-intensive and did not result in successful recruitment include the activities directed towards the GP medical centres. Comprehensive recruitment programs employ overlapping strategies simultaneously with ongoing assessment of recruitment rates. In our experience, and others direct mail-outs work best although rights to privacy must be respected. DOI: 10.1186/1471-2288-9-78 |
| Unson 2004 | Information on the characteristics of potential volunteers and their communities will enable readers to evaluate the applicability of recruitment methods used.  DOI: 10.1177/0898264304268588 |
| Cancer survivorship | |
| Cantrell 2012 | Additional research is needed on strategies to successfully recruit and retain older adolescents and young adult female survivors of childhood cancer in longitudinal intervention studies. DOI: 10.1188/12.ONF.483-490 |
| Cardiovascular disease | |
| Pribulick 2010 | Research studies need to be conducted in order to build a body of evidence for nursing interventions to reduce cardiovascular disease risk factors in rural women. A study is strengthened by a robust sample that provides power to statistical analysis. Without discussion of real-world experiences and appropriate and effective recruitment and retention strategies in nursing research, there is little chance of conducting research with appropriate power to build evidence-based practice.  PMID: 23641192 |
| Staffileno 2006 | This report summarizes existing recruitment and retention methods from the literature and describes how effective these strategies were in recruiting and retaining young, mildly hypertensive African American women to a physical activity intervention study. Multiple strategies, resources, and time were necessary to recruit and retain these women for the study. Among women enrolled, newspaper advertisements and flyers were the most effective recruiting strategies implemented (46% and 21%, respectively). Study retention was high (96%), which may have resulted from flexible scheduling, frequent contact, and a caring environment. Recruiting and retaining efforts need to be tailored o meet the needs of the target population.  DOI: 10.1097/00005082-200605000-00009 |
| Wilbur 2013 | Results suggest that provision of health assessment screening by study staff as part of recruitment is effective for minimizing attrition and also might be cost-effective.  DOI: 10.1002/nur.21550 |
| Wilbur 2001 | The findings also illustrate the importance of using multiple recruitment strategies to encourage midlife African American and Caucasian women to participate in intervention trials; exclusion of women with lower SES backgrounds or lower formal education level. DOI: 10.1002/nur.21550 |
| Infectious diseases | |
| Atherton 2007 | As in other studies, a key to attaining recruitment targets was the enthusiasm of the research team. Minority ethnic groups were probably under-represented, but understanding of participants was good. DOI: 10.1186/1745-6215-8-41 |
| Falcon 2011 | The successes of GRACE in enrolling a representative population of women were rooted in pretrial preparation, engagement of community advisors, enrollment quotas, choice of study sites and site support. Lessons learned from GRACE may be applied to future study design. Further focus on factors that influence discontinuation is warranted. DOI: 10.1089/jwh.2010.2504 |
| Fitch 2020 | REPRIEVE met its overall projected recruitment goal by using multiple, simultaneous strategies to specifically target a diverse population including minority subgroups. REPRIEVE benefited from the development of recruitment strategies with clear targets and communication of accrual targets to study teams.  DOI: 10.1080/25787489.2020.1733794 |
| Ivaz 2006 | For a study on a sensitive topic, two researchers recruiting women in groups after lectures may be a more effective and cost-effective way than individual recruitment by researchers working alone. DOI: 10.1093/fampra/cmi109 |
| Jones 2017 | Preliminary data suggest that the extent to which ad headlines and photos tap into authentic social experience, advertising on Facebook can extend geographic reach and provide a comparative sample to women recruited on-the-ground.  DOI: 10.1007/s10461-017-1797-3 |
| Wiemann 2005 | Strategies learned include: (1) Educate clinic staff on the rigors of study design; (2) Facilitate a team effort between clinical and research staff; modify recruitment procedures, as needed; (3) Provide prospective participants the option of enrolling by return appointment; (4) Anticipate a diminishing recruitment pool over time; (5) Set positive recruitment tone at the beginning of each clinic session; (6) Consider participants' mothers as important points of contact; (7) Match communication styles to participant contacts; and (8) Consider a variety of retention techniques. Together, these strategies helped to reinforce participant's commitment to the project, facilitated their attendance at interviews, and encouraged them to adhere to the treatment protocol. DOI: 10.1016/j.jpag.2005.09.006 |
| Interpersonal Violence | |
| Koziol-McLain 2016 | Print advertisements, website links, and networking were costly and inefficient methods for recruiting participants to a Web-based eHealth trial. Researchers are advised to limit their recruitment efforts to Web-based online marketplace and classified advertising platforms, as in the isafe case, or to social media. Online classified advertising in "Jobs-Other-volunteers" successfully recruited a diverse sample of women experiencing intimate partner violence. Preintervention recruitment data provide critical information to inform future research and critical analysis of Web-based eHealth trials. DOI: 10.2196/jmir.6515 |
| Mental health | |
| DeBar 2009 | Aggressive outreach and screening is likely not feasible for broader dissemination in everyday practice settings and recruits individuals with more similar demographic and clinical characteristics to those recruited through more abbreviated and realistic screening procedures than anticipated. DOI: 10.1016/j.cct.2009.02.007 |
| McDermott 2004 | These results reveal a difficulty in planning recruitment from a small population such as partially recovered anorexics. A small population's total pool size diminishes faster than it is replenished, suggesting that future studies of anorexia nervosa may recruit more successfully from many sites in a short period rather than at a few sites over a long period. DOI: 10.1002/eat.10231 |
| Vollert 2020 | Recruitment through media seems both more feasible and suitable to reach individuals in need of indicative prevention and should be part of a multimodal recruitment package. Future studies should be explicitly designed to investigate the impact of recruitment modality on reach and effectiveness including cost-effectiveness analyses. DOI: 10.1002/eat.23250 |
| Metabolic health | |
| Brown 2012 | The results indicate that a simple modification to a standard recruitment letter can have a meaningful impact on minority reach and recruitment rates. Practical implications include using ethnically-targeted, non-personalized direct mail letters and recruiting through friends/family at no additional cost. DOI: 10.1016/j.cct.2012.03.003 |
| Burns 2008 | The use of multiple strategies can enhance recruitment and retention of rural, older African American women into a research study. Strategies are most effective when they build a relationship of trust with participants and the community and make it easy and rewarding for women to participate. DOI: 10.1177/0145721708325764 |
| Griffin 2013 | Recruitment of overweight and obese Generation Y women for a clinical weight loss trial was difficult. Multiple strategies targeted at this age and gender group were required. Less rigorous selection criteria and reduced face-to-face intervention time may improve recruitment and retention rates into clinical trials for this age group.  DOI: 10.6133/apjcn.2013.22.2.16 |
| Johnson 2015 | After implementation of the plan by the study team, enrollment increased 78% and recruitment goals were met 16 months ahead of schedule. Participant retention and study drug adherence was 100%. We conclude that community engagement is essential to the development of an effective multifaceted plan to improve recruitment of underrepresented groups in clinical trials. DOI: 10.1111/cts.12264 |
| Kozica 2015 | Multiple program promotion strategies including communication, marketing and partnering, as well as mobilization of social networks and peer persuasion, enabled engagement of rural women into a healthy lifestyle program. These recruitment strategies are consistent with successful strategies utilized previously to recruit urban-dwelling women into lifestyle programs. Future engagement efforts in rural settings could be enhanced by hosting multiple sessions within existing socio-cultural networks and assuring participants that they will not need to share their personal health information with others in their community. DOI: 10.1186/s13063-015-0860-5 |
| Newlin 2006 | Results confirm previous findings that indicate that Black Americans may be successfully recruited into research studies at moderate rates when traditional recruitment methods are enhanced and integrated with more culturally sensitive methods. Lessons learned are considered. PMID: 17061753 |
| Sharp 2008 | Black women were successfully recruited using in-person community recruitment, e-mail, and community flyers within close proximity to the intervention site. Careful consideration should be given to the advantages and disadvantages of various recruitment strategies that might not generalize across studies. DOI: 10.1123/jpah.5.6.870 |
| Not specified | |
| Brown 2002 | The focus group discussions provided information on successful recruitment techniques employed by research recruiters that may be replicated across research studies. DOI: 10.1016/s1049-3867(01)00145-1 |
| Nutrition | |
| Gerace 1995 | Response rates were similar between the methods of addressing envelopes and among the three vehicles for the message, suggesting that the least costly method of mailing should be used. DOI: 10.1016/s0197-2456(95)00041-0 |
| Leonard 2014 | Recruiting young women into nutrition research is challenging. Use of social media enhances recruitment, while Email, phone and text message contact improves retention within interventions. Further research comparing strategies to optimise recruitment and retention in young women, including flexible testing times, reminders and incentives is warranted. DOI: 10.1186/1745-6215-15-23 |
| Smith 2007 | The addition of the website screening option was associated with a relative 22% increase in recruitment combined with reduced staff time required for screening, thereby increasing screening efficiency. Web-based options for clinical trial recruitment and screening in targeted populations may increase response rates while also reducing staff time. DOI: 10.1177/1740774506075863 |
| Partner health | |
| Akers 2018 | Topics covered include designing the study infrastructure to optimize recruitment and enrollment tracking, creating a Facebook presence via a fan page, designing ads that attract potential participants while meeting Facebook's strict requirements, and planning and managing an advertising campaign that accommodates the rapid rate of diminishing returns for each ad. DOI: 10.2196/jmir.9372 |
| **Women-specific study conditions** | |
| Cancer | |
| Albrecht 2013 | In this article the authors provide an overview of common recruitment challenges as well as the actual challenges encountered, procedures and strategies implemented to counter these challenges, while investigating the combined intervention of flaxseed oil, fasting, caffeine, and exercise in women with recurrent or multi-drug resistant stage III or IV ovarian cancer. DOI: 10.1016/j.apnr.2013.05.003 |
| Bailey 2004 | The actual recruitment strategies were expansion to five geographically distinct clinical sites, use of nurse practitioners focused primarily on patient issues, extremely flexible study hours and location, honorariums, support for transportation and childcare, and creativity in maintaining contact with study participants. With these strategies, 90% of eligible patients consented to participate in the study.  DOI: 10.1016/j.apnr.2003.12.002 |
| Bonilla | This study is an important first step to understanding the impact of returning study results among a population that is underrepresented in research. Returning the results of studies and understanding the impact of doing so is consistent with maintaining community involvement in all phases of research. The findings suggest that sharing aggregate research results in simple language yields few problems in participants’ understanding of the results and is viewed as important by participants.  DOI: 10.1186/s13063-021-05945-8 |
| Blumenthal 1995 | In conducting research of this type in low-income minority communities, special attention must be given to issues of recruitment and retention if the validity of the study is to be preserved. PMID: 7721592 |
| Brewster 2002 | A media-based recruitment strategy was effective for this single-visit cervical prevention study. This approach may be effective for recruitment of other low-income groups to clinical trials. DOI: 10.1006/gyno.2002.6592 |
| Choi 2016 | Themes were identified in relation to recruitment: personal networks, formal networks at churches, building on trust and respect, and facilitating a non-threatening environment. Themes were identified for retention: trust and peer support. Qualified, well-trained CHWs can recruit and retain hard-to-reach immigrant women in a randomized trial by using multiple culturally sensitive strategies.  DOI: 10.1097/FCH.0000000000000089 |
| Daley 2007 | The number of patients randomised was marginally lower than anticipated. We were able to identify and highlight valuable information for planning the recruitment of future trials involving similar populations. DOI: 10.1016/j.cct.2007.02.009 |
| Derose 2000 | It was found that an extended resource intensive period of relationship-building and community-based activities were necessary to conduct church-based programs effectively, particularly among older and ethnically diverse urban populations.  DOI: 10.1177/109019810002700508 |
| Fouad 2014 | Results indicate that volunteer CHAs can be trained to serve as research partners and be effective in improving the retention and adherence of minority and low-income women in clinical trials. DOI: 10.1016/j.cct.2004.03.005 |
| Germino 2011 | To successfully recruit busy, younger African American cancer survivors, it is important to use a multifaceted approach, addressing cultural and racial/ethnic barriers to research participation; bridging gaps across cultures and communities; including the role of faith and beliefs in considering research participation; recognizing the demands of different life stages and economic situations and the place of research in the larger picture of peoples' lives. Designs for recruitment and retention need to be broadly conceptualized and specifically applied.  DOI: 10.1007/s11764-010-0150-x |
| Gillan 2009 | In multicenter trials, monitoring of local recruitment protocols is required to ensure that each center can maximize accrual targets. DOI: 10.1258/jms.2009.009023 |
| Goodwin 2000 | Five lessons were learned during recruitment for this trial: (1) multicenter randomized trials of psychosocial interventions are feasible, even in very ill patients, (2) the use of a group intervention effectively increased the required sample size by 50%, (3) similarity of randomization rates suggests that generalizability of study results will probably be comparable to that of other randomized cancer trials, (4) multidisciplinary collaborations and involvement of experienced researchers facilitated enrollment, and (5) most challenges encountered in recruitment were similar to those seen in all clinical trial. DOI: 10.1016/s0895-4356(99)00148-1 |
| Heiney 2010 | Application of H-ARF led to successful recruitment in an RBT. The findings highlight three areas that researchers should consider when devising recruitment plans: absolute numbers versus recruitment rate, cost, and efficiency with institutional review board–approved access to protected health information.  DOI: 10.1188/10.ONF.E160-E167 |
| Irwin 2008 | Findings from this study will provide useful information for investigators who are conducting exercise trials in cancer populations, clinicians who are treating women diagnosed with breast cancer, and exercise professionals who are developing community-based exercise programs for cancer survivors. DOI: 10.1002/cncr.23446 |
| Maxwell 2005 | We found that a personal invitation from either a female project liaison, a friend, or the Filipino project director were all successful strategies that resulted in over 80% attendance at an educational session that was offered as part of the study. Although non-attendees did not differ from attendees in demographic characteristics, they expressed significantly more barriers to participating in a health study. Attendance at the group session was a significant predictor of retention in the study. We were able to conduct telephone follow-up surveys among 88% of enrollees at 12 month follow-up and 76% at 24 month follow-up. Results and implications are discussed in the hope that they may facilitate future participation of Filipinos and other Asian immigrants in research. DOI: 10.1007/s10900-004-1956-0 |
| Menon 2008 | Planning and trial management are as important as trial design and require equal attention from senior investigators. Successful recruitment needs constant monitoring by a committed proactive management team that is willing to explore individual solutions for different centres and use central resources to improve local recruitment. Automation of trial processes with web-based trial management systems is crucial in large multicentre randomised controlled trials. Recruitment can be further enhanced by using information videos and group discussions. DOI: 10.1136/bmj.a2079 |
| Ott 2006 | Tracking recruitment efforts in large clinical trials should be ongoing, site-specific, and cost-effective. Changes incorporated early in the recruitment phase addressed unique aspects of rural versus metropolitan areas and resulted in near achievement of accrual goals. DOI: 10.1097/00002820-200601000-00004 |
| Pinto 2021 | The highest randomization rates were obtained via targeted mailings (88.2%) and the lowest via brochure/flyer (0.4%)…There were statistically significant differences in costs between recruitment methods (p<0.001) with lowest (non-negligible) cost being targeted mailings. The current RCT of breast cancer survivors successfully recruited and retained participants, highlighting the need to combine recruitment methods to achieve accrual goals. Recruitment methods differed substantially in their cost and their ability to attract individuals who would ultimately be randomized.  DOI: 10.1016/j.cct.2021.106285 |
| Russell 2008 | Four themes emerged from focus group discussions with community agency providers and research team members. These themes were (1) going to the gatekeepers; (2) knowing the culture; (3) location is everything; and (4) protocols, policies, and possibilities. A checklist of actions that nurse-researchers could consider to increase African American women's participation in community trials is provided. DOI: 10.1016/j.apnr.2006.05.001 |
| Santoyo Olsson 2019 | Spanish-speaking Latinas with non-metastatic breast cancer were recruited by community recruiters. Of 231 women approached. 24% refused, 10% were ineligible and 153 (66%) were randomized to the intervention or a wait-list control group....Applying the Transcreation Framework to engage stakeholders in designing community-based RCTs enhanced congruence with community contexts and recruitment of this vulnerable population. DOI: 10.1371/journal.pone.0224068 |
| Shuhatovich 2005 | The recruitment method that resulted in the most contacts was newspaper reportorial coverage and advertising, followed by family and friends, then television news coverage. The most cost-effective method for finding eligible women who attend the research appointment is word of mouth from a family member or friend. Recommendations are given for maximizing the efficiency of recruitment for cervical cancer screening trials. DOI: 10.1016/j.ygyno.2005.07.093 |
| Stratton 2015 | The availability of a large number of potential participants from the telecolposcopy network increased recruitment to this clinical trial by 85% over other traditional means of recruitment. The telecolposcopy network is not only a means of providing a gynecological service to women who otherwise would forego care but also a novel and valuable resource in recruiting participants for a clinical trial. DOI: [10.1177/1740774514566333](https://doi.org/10.1177/1740774514566333) |
| Swaine 2011 | It is possible to recruit community-dwelling women with intellectual disabilities into randomised controlled trials at relatively high participation rates. Recruiting women who have guardians poses additional challenges for researchers.  DOI: 10.1111/j.1365-2788.2011.01399.x |
| Sturgeon 2018 | The WISER Survivor trial faced multiple recruitment challenges and utilized unique strategies to successfully enroll minority breast cancer survivors into a lifestyle intervention. DOI: 10.1002/cncr.30935 |
| Tanjasiri 2015 | Although CBPR improves the cultural competence and relevance of study activities for ethnically diverse populations, selected past research shows that it does not ensure that such designs overcome all of the unique challenges in ethnically diverse communities. PI-specific organizational recruitment and individual retention is influenced by study issues and cultural factors in each community.  DOI: 10.1353/cpr.2015.0067 |
| Vogsen 2020 | Involving patients as partners in the research team resulted in major changes to the participator information material and contributed to higher-than-expected patient recruitment and retention. Furthermore, we observed a positive change of attitude amongst the researchers towards patient involvement in the research process.  DOI: [10.1186/s40900-019-0174-y](https://doi.org/10.1186/s40900-019-0174-y) |
| Cancer prevention | |
| Zhu 2000 | Because the single (relationship status) constitute 75% of African-American women aged 65 and older, and the incidence and mortality of cancer are especially high in elderly African Americans, our experiences are encouraging for cancer prevention and control research in the population. PMID: 10976173 |
| Contraception | |
| Rdesinski 2008 | Two hundred and forty-five women were identified in recruiting and enrolling 103 study participants involving 1,232 contact-attempts. Self-referral had the highest ratio of referrals to enrollees (55.6%), while this ratio was the lowest for community outreach (33.3%). Retention activities succeeded in maintaining over 90% of the sample. Ninety-two percent of English-speaking participants completed the study versus 79% of Spanish-speaking participants. The time expenditure per enrollee was 10.4 hours for recruitment and 1.2 hours for retention, with an estimated cost per enrollee of $324.03 for recruitment and $39.14 for retention. More retention activities were required to maintain women in the comparison group than in the intervention group. DOI: 10.1353/hpu.0.0016 |
| General Women’s Health | |
| Larkey 2002 | Embajadoras were more successful at referral and enrollment than untrained Hispanic women and more successful at enrollment than un- trained Anglo controls. Embajadoras were also found to distribute significantly more brochures than control groups. Therefore, a culturally aligned training program to encourage current Hispanic participants in a clinical trial to advocate the study to others may be an effective way to boost referrals and enrollments. Other potential influences on enrollment or referral success could not be determined due to the small sample size. Further study is needed to examine the best methods to encourage enrollment for women referred to the study. DOI: 10.1016/s0197-2456(02)00190-3 |
| Pastore 2009 | Posters/flyers and direct mailings proved to be the most successful recruitment methods for this CAM study. Active recruitment with multiple methods was needed for continual enrollment. DOI: 10.1016/j.ctim.2009.03.004 |
| Sweet 2008 | The Center for Research in Reproduction at Meharry set out to recruit a large number of African American women volunteers of reproductive age into clinical trials. The experience, of recruiting volunteers from the African American community for clinical trials in the Meharry Medical College/Pennsylvania State University (MMC/PSU)'s Cooperative Center for Research in Reproduction at Meharry, is presented.  DOI: 10.1016/j.cct.2007.11.003 |
| Gynecologic conditions | |
| Bachour 2017 | In this clinical trial, mass mailing was the most effective recruitment method. Race of participants enrolled in a provoked vulvodynia trial was related to the recruitment method. DOI: 10.1177/1740774516663461 |
| Benham 2021 | For this trial, each method was important for recruiting inactive women with PCOS because no participant reported learning about the trial through more than one method. Unpaid advertisements and Facebook advertisements helped recruit the  largest number of participants in the trial, the former resulting in a higher cost per participant than the latter. DOI: 10.2196/25208 |
| Benoit-Piau 2020 | The study findings revealed that e-recruitment is a valuable recruitment method because of its comparable efficiency and cost-effectiveness to health professional referrals and conventional methods, respectively. DOI: 10.1016/j.jsxm.2020.04.005 |
| Blödt 2016 | Our results contribute to the ongoing discussion of the impact of financial compensation on research participants' assessment of risk. The interviewed women considered all research participants able to make their own choices regarding trial participation, even in the face of financial compensation or payment of study participants. Furthermore, the importance of clinical trials providing new treatments that could change medical practice might be an overlooked reason for trial participation and could be used in future recruitment strategies.  DOI: 10.1136/bmjopen-2016-012592 |
| Breitkopf 2011 | In general, women viewed reimbursement as a benefit to research participation, the amount of which should reflect time, the inconvenience to the research subject, and the potential for unknown risks in the short- and long-term. They believed reimbursement should take into account the degree of risk of the study, with investigations of experimental products offering greater reimbursement. Women believed that monetary reimbursement is unlikely to coerce an individual to volunteer for a study involving procedures or requirements that they found unacceptable. The results of this study can be used to provide guidance to those planning and evaluating reimbursement for research participation. DOI: 10.1525/jer.2011.6.3.31 |
| Brubaker 2013 | Participants in both groups believed that their physicians were the best source of information about clinical trials yet felt that other sources of trial information were important. Financial compensation was not a primary motivating factor for PFD trial enrollment but was, however, cited as an important consideration...This study identified central themes guiding successful recruitment to and retention in PFD-related trials and provided insight regarding strategies that may guide future trials.  DOI: 10.1007/s00192-012-1824-x |
| Cambron 2001 | The most effective recruitment methods were newspaper advertisements, community referrals, and radio advertisements; the least effective methods were public television and local posters. DOI: [10.1067/mmt.2001.112567](https://doi.org/10.1067/mmt.2001.112567) |
| Dickson 2013 | Our article reflects on the methodological challenges of recruiting to a multi-centre RCT in a UK gynaecology setting. Effective interventions included increasing the number of recruiting centres and providing collaborator incentives. Barriers to recruitment included fewer eligible women than anticipated, patient's preference to be allocated to the treatment group, lack of support staff, and variations in approval systems and GP referral procedures. To improve the evidence base on clinical trial recruitment, trialists need to publish their experiences and lessons learned. Future RCTs should evaluate, where possible, the effect of strategies designed to improve recruitment and retention. DOI: 10.1186/1745-6215-14-389 |
| Goode 2008 | Questionnaires were completed by 23 physician investigators and 11 nurses or coordinators (92% response rate). Respondents indicated it was more difficult to recruit older research participants (32%), obtain informed consent (56%), and retain participants to study completion (50%). Challenges to recruitment included caregiver involvement in the decision to participate and participant comorbidities. Perceived barriers to retention were transportation, caregiver availability, and participant fatigue. Data quality was challenged by sensory and cognitive impairment, resulting in a change from telephone interviews to in-person visits in the Colpocleisis study. Older participants did not have higher dropout rates than younger participants. There were no differences in missed in-person visits or telephone interview rates between age groups. DOI: 10.1016/j.jamcollsurg.2008.03.012 |
| Infectious disease | |
| Bleidorn 2015 | To optimize recruitment conditions for further clinical trials on acute and common conditions in family medicine, the following key issues should be considered: emphasizing patients' personal benefit, featuring patient relevant trial topics, providing a maximum of safety, keeping effort by trial procedures comfortable.  DOI: 10.3205/000221 |
| Menopause | |
| Butt 2010 | Newspaper advertisements were the most successful method to recruit postmenopausal women into a community-based, primary care RCT.  DOI: 10.1016/j.cct.2010.06.003 |
| Folmar 2001 | Enriched sources of recruitment yielded higher percentages of enrolled participants than nonenriched sources. Both types of source resulted in demographically similar participants. Costs of community-based recruitment were less than hospital-based recruitment; however, screening costs were higher. Overall, screening and recruitment averaged $2508 per randomized participant.  DOI: 10.1016/s0197-2456(00)00117-3 |
| Hemminki 2004 | Blinding decreased women's interest in joining a long-term preventive trial. Women's reasons for joining the trial were not influenced by blinding.  DOI: 10.1016/j.jclinepi.2004.04.009 |
| Fouad 2004 | Of those who expressed initial interest in WHI, African Americans had the highest randomization yields in the DM component and Hispanics had the highest in the HRT component (15.2% and 10.2%, respectively). Overall, mass mailing was the greatest source of randomized participants. In addition, minority clinics found community outreach, personal referrals, and culturally appropriate recruitment materials particularly effective recruitment tools. For minority recruitment, our findings suggest that the key to high yield is reaching the target population through appropriate recruitment strategies and study information that get their attention. Also, once minority subjects are reached, they tend to participate.  DOI: 10.1016/j.cct.2004.03.005 |
| Lindenstruth 2006 | A variety of targeted recruitment strategies are required to ensure a diverse response to advertisements and promotions. Given the extra time and effort needed to recruit minorities, it is essential that researchers include adequate resources to cover the cost of recruitment in their budgets. PMID: 17061750 |
| Moody 1995 | Current health periodicals geared toward nurse practitioners contain advertisements that recruit nurse practitioners to serve as clinical trial coordinators. In this health science center, there are five nurse practitioners who are employed in this role. Students in the master’s program report that they were sent back to graduate school by their employers to learn how to do research. It appears that clinical research is now more valued by students and clinicians. The use of advanced nurse practitioners in the role of clinical trial coordinator is natural because of their education and preparation, high level of interpersonal skill, physical assessment skills, ability to perform diagnostic tests and interpret results for screening, and organizational skills. This may emerge to be a key role for master’s-prepared nurse practitioners who have a strong foundation in research. DOI: 10.1111/j.1745-7599.1995.tb01116.x |
| Paine 2008 | Conducting group seminars with potential participants may be a useful strategy for maximising recruitment from general practice, by increasing patient information and reducing a research team's workload. DOI: 10.1186/1745-6215-9-5 |
| Panjari 2008 | Despite ongoing interest by women to participate in research for therapies to treat low libido, concerns about the use of any hormonal treatment and the time poverty experienced by many women at midlife present new barriers to recruitment and need to be considered in assessing the feasibility of studies in this field.  DOI: 10.1089/jwh.2007.0732 |
| Tworoger 2002 | The proportion of respondents eventually randomized did not differ by recruitment strategy (mail, media, other). Our study indicates that bulk mail may be more cost-effective than first-class mail for recruitment into intervention trials and that older women are willing to participate in such studies. PMID: 11815403 |
| Waltman 2019 | Recruitment could be more costly and time-consuming than anticipated. Recruitment using direct-targeted mailings, such as provider letters and postcards, were successful in our study and have been effective in previous studies reviewed. Facebook was successful for recruitment in our study and may continue to be useful for recruitment in the future, as the number of women accessing Facebook continues to increase. DOI: 10.1097/NNR.0000000000000356 |
| Peripartum | |
| Barnett 2012 | Qualitative assessment of the participants' study experience suggesting that high retention was due to strong rapport with participants, short interviews requiring little time commitment, and participants' perception of the study as informative, provides further evidence of our approach's effectiveness. Logistical protocol procedures and staff management strategies relating to successful recruitment/ retention are provided to propose a practical, cost-effective and translational recruitment-retention plan for other researchers to adopt. DOI: 10.1016/j.cct.2012.06.005 |
| Barrera 2014 | The Internet is an effective method for reaching an international sample of pregnant women interested i online interventions to manage changes in their mood during the perinatal period. To increase efficiency, Internet advertisements need to be monitored and tailored to reflect the target population's conceptualization of health issues being studied. DOI: 10.2196/jmir.2999 |
| Brown 2015 | Results from this randomized study appear to suggest that recruitment letters with diabetes health risk information targeted to recipients' race/ethnicity may improve one metric of clinical trial participation among Latina women who prefer Spanish, but not English. Larger experimental studies, incorporating input from diverse participant stakeholders, are needed to develop evidence-based minority recruitment strategies.  DOI: 10.1177/1740774514568125 |
| Carpenter 2016 | Our experience of recruitment and retention of pregnant women into a physical exercise intervention study has provided useful insight into these processes. We designed a study that enabled participants to have the freedom to choose which arm of the randomised trial they took part in. This encouraged participation amongst women and may have reduced the rate of dropout in our study. It therefore should be considered for future trials of a similar nature. DOI: 10.3109/01443615.2015.1049988 |
| Catherine 2020 | Our retention results are encouraging given that participants were experiencing considerable socioeconomic disadvantage. Standardized retention planning and reporting may therefore be feasible for health research in general, using the framework we have developed. Use of standardized retention protocols should be encouraged in research to promote consistency across diverse studies, as now happens with RCT and SR protocols. Beyond this, successful retention approaches may help inform health policy-makers and practitioners who also need to better reach, engage and retain underserved populations. DOI: 10.1186/s13063-020-04328-9 |
| Coleman-Phox 2013 | The narrow window of eligibility for enrolling early-stage pregnant women in a group intervention presents obstacles. In-person recruitment was the most successful strategy; establishing close relationships with providers, clinic staff, social service providers, and study participants was essential to successful recruitment and retention. DOI: 10.5888/pcd10.120096 |
| Daniels 2012 | The consent and retention rates of our sample of first-time mothers are comparable with or better than other similar studies. The recruitment strategy used allowed for detailed information from non-consenters to be collected; thus, selection bias could be estimated. Recommendations for future studies include being able to contact participants via mobile phone (particularly text messaging), offering home visits to reduce participant burden and considering the use of financial incentives to support participant retention. DOI: 10.1186/1479-5868-9-129 |
| Ekambareshwar 2018 | Despite some challenges in recruiting pregnant women to an infant obesity prevention programme, some of the facilitators in recruitment included mode of delivery of the intervention programme via telephone calls or text messages, the minimal effort required for women to participate, organisational support from the lead site, and recruiters' knowledge of and commitment towards the trial.  DOI: 10.1186/s13063-018-2871-5 |
| El-Khorazaty 2007 | We conclude that with targeted recruitment and retention strategies, minority women will participate at high rates in behavioral clinical trials. We also found that women who drop out are different from women who are lost to follow-up and require different strategies to optimize their completion of the study. DOI: 10.1186/1471-2458-7-233 |
| Homer 2000 | The STOMP trial has provided our team with invaluable experience in conducting research in a multicultural context. The strategies discussed can be used in research in other contexts to ensure that the voices of all our community are represented in our research. This is particularly important when the findings of research are used to change practice or implement new models of care. DOI: 10.1054/midw.2000.0230 |
| Houghton 2018 | Our findings do not support a single pathway to consent in the context of an obstetric emergency. Women understand that consent to research in an emergency is complex. Clinicians' skills in considering the clinical, ethical, and emotional aspects within the context of the clinical emergency can hamper or promote women's satisfaction. DOI: 10.1111/1471-0528.15333 |
| Kenyon 2006 | Women gave prominence to the socioemotional aspects of their interactions with healthcare professionals in making decisions on trial participation. Comments on the quality of written and spoken information were generally favourable, but women's accounts suggest that the stressful nature of the situation affected their ability to absorb the information. Women generally had poor understanding of trial design and practices. The main motivation for trial participation was the possibility of an improved outcome for the baby. The second and less prominent motivation was the opportunity to help others, but this was conditional on there being no risks associated with trial participation. In judging the risks of participation, women seemed to draw on ''common sense'' understandings including a perception that antibiotics were risk free. DOI: 10.1136/qshc.2005.015636 |
| Le 2008 | The recruitment rates (i.e., the number of participants who met eligibility criteria, consented, and randomized into the study) were 70% in the U.S. and in Mexico. Issues and recommendations related to recruiting Hispanic women into preventive intervention trials for postpartum depression are discussed.  DOI: 10.1007/s00737-008-0009-6 |
| Leavitt 2017 | This study demonstrates success recruiting pregnant smokers using text message. Future studies should consider building on this approach for recruiting high-risk populations. DOI: 10.1007/s13142-016-0450-4 |
| Lesher 2015 | Provider and clinic-based recruitment was the most effective and cost-efficient method of recruitment in a preconception intervention study of reproduction among women. DOI: 10.1111/ppe.12177 |
| Lopez 2008 | A proactive recruitment strategy (telephoning women whose phone numbers were purchased from a marketing firm) was ultimately much more successful than a variety of reactive strategies (advertisement, press releases, direct mail, web placement, healthcare provider outreach). DOI: 10.1080/14622200701704962 |
| MacLachlan 2021 | Recruitment resulted from a combination of all three strategies. Our reflections on the successes and challenges of these strategies highlight the need for recruitment strategies to be flexible to adapt to complex interventions and real-world challenges. These findings will inform future research in similar hard-to-reach populations.  DOI: 10.1186/s13063-021-05348-9 |
| Martin 2013 | A recruitment strategy designed to incorporate and respond to patient feedback improved recruitment of Black and Latina women to a clinical trial.  DOI: 10.1353/hpu.2013.0125 |
| Nicklas 2016 | Our multilevel approach allowed us to successfully meet our recruitment goal and recruit a representative sample of women with recent GDM. We believe that our most successful strategies included using a dedicated in-person recruiter, integrating recruitment into clinical flow, allowing for flexibility in recruitment, minimizing barriers to participation, and using an opt out strategy with providers. Although the majority of women were recruited while pregnant, women recruited in the early postpartum period were more likely to present for the first study visit. Given the increased challenges of recruiting postpartum women with GDM into research studies, we believe our findings will be useful to other investigators seeking to study this population. DOI: 10.1007/s10995-015-1825-8 |
| Oakley 2003 | Procedures can be developed for recruiting people with diverse cultural backgrounds to take part in research. This helps to address the issue of possible bias in generalizing research findings by increasing external validity and respects the ethic that everyone should have the right to be eligible for inclusion in research.  DOI: 10.1080/13557850303554 |
| Paquin 2021 | Although we sent letters and emails in roughly equal proportion by urbanicity and race/ethnicity, we found significant differences in enrollment across demographic subgroups. Controlling for race/ethnicity and urbanicity, we found that direct-mail letters and emails were effective recruitment methods. The enrollment rate among women who were sent a recruitment letter was 4.1%, and this rate increased to 5.0% among women who were also sent an email invitation.  DOI: 10.1111/cts.12950 |
| Park 2007 | A recruitment strategy using a health plan's centralized system was more efficient than a practice-based recruitment strategy at identifying potential study participants, but less efficient at generating study participants from the referrals received. Importantly, participants recruited by the two strategies differed by socioeconomic, but not cessation-related, characteristics. To date, recruiting pregnant smokers into intervention studies remains resource intensive and time consuming. Participant identification and recruitment will be greatly enhanced by health system innovations such as implementation of electronic medical records. DOI: 10.1016/j.ypmed.2006.10.008 |
| Peindl 2003 | These referrals yielded relatively high rates of participation. However, other methods were differentially successful for the two studies: media appearances and advertising resulted in almost 50% of the screening calls for the prevention study, whereas mass mailings and other promotional materials were more effective for the treatment study. Those materials contributed 44% of screening calls for the treatment study. Our conclusions were that women were most likely to enter the research studies when referred by an obstetrician or other professional. DOI: 10.1016/s0022-3956(02)00086-9 |
| Phillippi 2018 | E-consent is feasible and easy to use with pregnant women and may expedite enrollment of a representative sample. DOI: 10.1016/j.jogn.2018.04.134 |
| Phipps 2013 | Intrapartum research is associated with low rates of recruitment and these rates may be improved by asking women to provide informed consent during labor rather than the antenatal period. It is important to consider ways to facilitate randomized controlled trials involving women in labor to advance evidence-based care in this environment. This should include prospective research that seeks to define the best approach to patient recruitment. DOI: 10.1111/aogs.12243 |
| Pollak 2006 | This paper describes recruitment efforts for a multiclinic trial to test the effectiveness of NRT use in addition to behavioral therapy in promoting cessation during pregnancy. The biggest challenge is recruiting sufficient numbers of pregnant women. This paper discusses specific obstacles for recruitment and solutions. Knowing the potential pitfalls to recruiting pregnant women into these trials can lead to better studies and thus improved outcomes. DOI: 10.1080/14622200600789882 |
| Price 2019 | By taking a quality improvement approach, supported by sufficient resourcing and flexible research processes, it is possible to recruit and retain a large cohort of women experiencing adversity who are typically missed or lost from longitudinal research. DOI: 10.1186/s12913-019-4698-5 |
| Ramos-Gomez 2008 | Recruitment and retention efforts for pregnant Hispanic women should place heavy emphasis on culture as ethnicity remained the only borderline significant predictor in post randomization retention. DOI: 10.1177/1740774508093980 |
| Salihu 2015 | Our positive experience will be of utility to other researchers globally. Our findings have far-reaching implications as the socio-ecological model approach is adaptable to developed and developing regions and has the potential to increase recruitment and retention of hard-to-reach populations who are typically under-represented in clinical trials. PMID: 27621990 |
| Shere 2014 | Clinicians and scientists recruiting for clinical studies should learn how to use online social media platforms to improve recruitment rates, thus increasing recruitment efficiency and cost-effectiveness. DOI: 10.1371/journal.pone.0092744 |
| Smith 2006 | It is hoped that the experiences described here will give some insight to recruitment and implementation strategies. There is a need for more systematic research and evaluation of these strategies, and dissemination of these findings to assist with successful implementation of trials. DOI: 10.1016/j.ctim.2005.07.004 |
| Smyth 2009 | Women were largely positive about participation in the trial and its follow-up, but still reported ways they believed the study could have been improved, such as more information, given earlier, which also has implications for clinical care.  DOI: 10.1111/j.1523-536X.2009.00326.x |
| Stendell-Hollis 2011 | Researchers conducting studies with lactating women may consider exclusive breastfeeding as a study inclusion criterion to prevent high attrition rates or include additional breastfeeding support to study participants. DOI: 10.1016/j.cct.2011.03.007 |
| Usadi 2015 | Physician referral was the most successful recruitment strategy. Radio ads and the internet were the next most successful strategies, particularly for women of limited income. Ancillary clinical sites were important for overall recruitment.  DOI: 10.1016/j.cct.2015.09.010 |
| Velott 2008 | Successful recruitment of typically hard-to-reach women, such as low-income rural women, is possible through implementation of a triangular recruitment approach in local communities. DOI: 10.1016/j.whi.2008.02.002 |
| Vignato 2019 | Mobile health applications are innovative venues for recruiting research participants.  DOI: 10.1007/s00737-018-0894-2 |
| Webb 2010 | Findings challenge beliefs that low income and minority women are averse to enrolling and continuing in clinical trials or community studies.  DOI: 10.1186/1471-2288-10-88 |
| Urogynecology | |
| Agnew 2013 | The recruitment rate for a continence promotion trial among older women known to be eligible and attending workshops hosted by local community organisations was high (44%). Strategies are needed to bolster community organisations' involvement in health promotion trials in general and for continence issues in particular.  DOI: 10.1177/1740774512460144 |
| Messer 2006 | The mass mailing strategy was an effective means of recruiting a representative sample of women, aged 55-80. Short falls and recommendations for successful community sample recruitment strategies for clinical trials in older adult women are elaborated upon. DOI: 10.1007/s11255-006-0018-1 |
| Van der Worp 2020 | Samples recruited through the media and through case identification were largely comparable. Therefore, recruitment through the media may be a viable alternative to recruitment through primary care. This may be especially relevant for research on eHealth treatment for conditions with which patients experience barriers when seeking health care. DOI: 10.1016/j.jclinepi.2019.12.001 |

*Text copied verbatim from indicated citation.
